# Dementia blood biomarkers in the context of post‐stroke cognitive outcomes: Systematic review and evidence synthesis

**DOI:** 10.1002/alz.71653

**Published:** 2026-07-06

**Authors:** Hing Tim Fung, Olivia Burton, Mara Bortnowschi, Paul M Matthews, Laura M Parkes, Henrik Zetterberg, Atticus H Hainsworth, Fatemeh Geranmayeh

**Affiliations:** ^1^ Department of Brain Sciences Imperial College London London UK; ^2^ Imperial College Healthcare NHS Trust London UK; ^3^ UK Dementia Research Institute Centre Imperial College London London UK; ^4^ Rosalind Franklin Institute Didcot UK; ^5^ School of Health Sciences Faculty of Biology, Medicine and Health University of Manchester Manchester UK; ^6^ Geoffrey Jefferson Brain Research Centre University of Manchester, Manchester Academic Health Science Centre Manchester UK; ^7^ Department of Psychiatry and Neurochemistry Institute of Neuroscience and Physiology Sahlgrenska Academy at the University of Gothenburg Mölndal Sweden; ^8^ Clinical Neurochemistry Laboratory Sahlgrenska University Hospital Mölndal Sweden; ^9^ Department of Pathology and Laboratory Medicine University of Wisconsin School of Medicine and Public Health Madison Wisconsin USA; ^10^ Wisconsin Alzheimer's Disease Research Center University of Wisconsin–Madison School of Medicine and Public Health Madison Wisconsin USA; ^11^ Department of Neurodegenerative Disease UCL Institute of Neurology, Queen Square London UK; ^12^ UK Dementia Research Institute at UCL London UK; ^13^ Centre for Brain Research Indian Institute of Science Bangalore India; ^14^ City‐St George's University of London London UK; ^15^ Department of Neurology St George's University Hospitals NHS Foundation Trust London UK

**Keywords:** amyloid beta, blood‐based biomarkers, cognitive impairment, glial fibrillary acidic protein, hemorrhagic stroke, ischemic stroke, neurofilament light chain, placental growth factor, stroke, systematic review, tau protein

## Abstract

**INTRODUCTION:**

The prognostic value of emerging dementia‐related blood‐based biomarkers for post‐stroke cognitive impairment is poorly understood. We addressed this critical gap in this systematic review.

**METHODS:**

Four databases were searched in March 2025 for studies of neurofilament light (NfL), glial fibrillary acidic protein (GFAP), amyloid beta (Aβ), tau, and placental growth factor (PlGF) in relation to post‐stroke cognitive outcomes. Risk of bias, narrative synthesis, and meta‐analysis were performed.

**RESULTS:**

Eighteen studies were included, eleven assessing NfL. Meta‐analysis of four studies (*n *= 2020) showed higher acute NfL was associated with worse cognition at 1–6 months (*Z *= −0.518, 95% confidence interval [CI] −0.684 to −0.351). Baseline GFAP was associated with worse cognition longitudinally, whereas results for amyloid and tau species were inconsistent between studies. Risk of bias was high.

**DISCUSSION:**

NfL and GFAP show the most consistent associations with post‐stroke cognition, particularly acutely. Evidence for amyloid and tau was inconsistent between studies, and PlGF remains unexplored.

## Highlights

- Dementia‐related blood biomarkers and post‐stroke cognition were assessed
- The systematic review identified 18 studies
- Higher early blood neurofilament light (NfL) predicted poorer cognition within 1–6 months after stroke
- Evidence for blood amyloid beta and tau associations with cognition is inconsistent
- Blood glial fibrillary acidic protein (GFAP) shows potential as a biomarker of post‐stroke cognitive status

## 1 INTRODUCTION

Vascular disease is the second most common contributor to dementia^1^ and vascular processes are one of the earliest drivers of Alzheimer's disease (AD).^2^ Of note, a substantial proportion of dementia risk is attributable to modifiable factors, many of which are cerebrovascular in nature.^3^ Stroke remains a leading cause of vascular dementia^4^ and post‐stroke cognitive impairment (PSCI) is common affecting up to 70%–80% of survivors.^5^ Within 1 year, ≈8% of patients with mild stroke develop post‐stroke dementia (PSD), rising to 34% in those with more severe events.^6^ With over 94 million stroke survivors worldwide,^7^ there is a pressing need to identify reliable biomarkers of post‐stroke cognitive decline. Such biomarkers would enable early risk stratification, support patient selection, and provide objective endpoints for clinical trials targeting cognitive recovery, potentially reducing trial duration, cost, and uncertainty. Identifying these markers is therefore important both for understanding post‐stroke pathology and for guiding interventions to preserve cognition and prevent cognitive decline.

Blood‐based biomarkers (BBMs) have transformed dementia research, particularly in AD, enabled by ultra‐sensitive detection technologies.^8^ These biomarkers capture diverse neuropathological processes, including neuroaxonal injury (neurofilament light [NfL]),^9^ astroglial activation (glial fibrillary acidic protein [GFAP]),^10^ and AD‐specific pathology including amyloid beta (Aβ40, Aβ42),^11^, ^12^ total‐tau (t‐tau),^13^ and phosphorylated tau (p‐tau) species (p‐tau181 and p‐tau217).^14^, ^15^

These dementia‐related BBMs have been studied extensively in dementia cohorts but less frequently in cohorts with concomitant cerebrovascular disease (CVD). Where examined, their performance appears altered by vascular pathology. For instance, plasma GFAP detects amyloid pathology in cognitively impaired individuals, but its accuracy diminishes with increasing white matter hyperintensity (WMH) burden.^16^ Blood placental growth factor (PlGF), a marker of endothelial dysfunction,^17^ distinguishes individuals with cerebrovascular pathology burden (WMHs, microbleeds) from those without cerebrovascular pathology burden in AD cohorts,^18^ and separates vascular cognitive impairment from cognitively healthy individuals.^19^ In contrast, p‐tau181, p‐tau217, and the Aβ42/Aβ40 ratio appear to remain robust in the presence of WMH burden, supporting their utility for identifying AD‐related pathology despite coexisting CVD.^16^, ^20^ Furthermore, NfL may have the potential to stratify individual and combined AD and CVD pathological contributions to cognitive impairment.^21^

Although these markers have been characterized in dementia and CVD populations, their performance in the biological context of stroke remains poorly defined. Most existing stroke studies have focused on non‐cognitive outcomes: experimental animal models have shown that stroke alters Aβ production and Aβ clearance,^22^, ^23^ whereas human studies have linked Aβ levels to unfavourable post‐stroke neurological and functional outcomes, measured using the National Institutes of Health Stroke Scale (NIHSS) and modified Rankin Scale (mRS).^24^ Blood‐based brain‐derived tau (BD‐tau), a marker of neurodegeneration, has been associated with poorer global functional outcome after stroke.^25^ Among these BBMs, NfL has been most studied extensively after stroke. Serum NfL concentrations peak between 7 days and 1 month post‐stroke and are associated with stroke severity, infarct volume, and poorer long‐term functional outcomes.^26^, ^27^, ^28^, ^29^, ^30^ Plasma GFAP concentrations typically peak within hours following a stroke,^31^ demonstrate predictive utility for neurological impairment (NIHSS) and functional disability (mRS),^32^ and differentiate hemorrhagic and ischemic sub‐types.^33^ Emerging evidence also implicates vascular‐specific biomarkers such as PlGF in predicting first‐time cardiovascular events, including stroke,^34^ and in cognitive outcomes in small vessel disease (SVD) related vascular dementia.^35^

Despite these insights, few studies have investigated whether these BBMs are associated with post‐stroke cognitive trajectories rather than neurological or functional outcomes alone. Ongoing longitudinal studies aim to clarify their role in predicting cognitive change after stroke.^36^, ^37^, ^38^ This systematic review synthesizes current evidence on dementia‐related BBMs and their relationship to post‐stroke cognitive outcomes, evaluating their potential for prognostic stratification and mechanistic insight into cognitive trajectories.

## 2 METHODS

### 2.1 Search strategy

**FIGURE 1:**
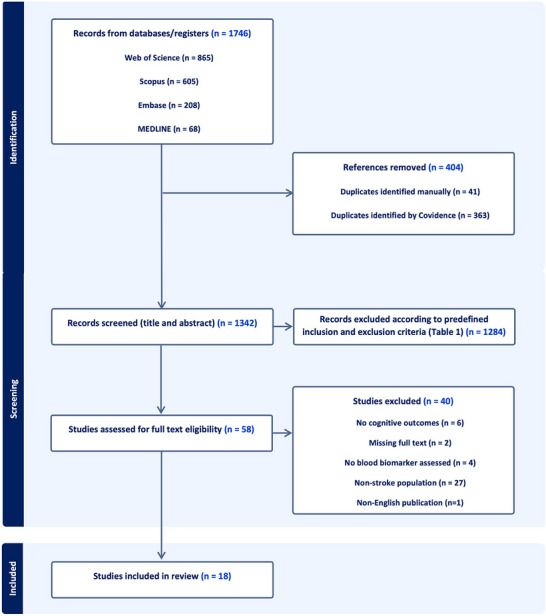
PRISMA (Preferred Reporting Items for Systematic reviews and Meta‐Analyses) chart illustrating the study selection process. Note. The figure includes the total number of reports identified and the reasons for exclusion. (See Table 1 for inclusion and exclusion criteria.)

This systematic review followed the Preferred Reporting Items for Systematic reviews and Meta‐Analyses (PRISMA) guidelines.^39^ A comprehensive search of MEDLINE, EMBASE, Web of Science, and Scopus was performed on March 13, 2025. Non–English‐language studies were excluded. Search terminology encompassed stroke, cognitive impairment (PSCI and vascular dementia), and BBMs of interest: NfL, GFAP, tau (phosphorylated and total), Aβ (40 and 42), and PlGF. To ensure relevance to peripherally circulating biomarkers, sample types (blood, serum, and plasma) were also included. Full search strategies are provided in Supplementary Materials 1. Identified studies were imported into Covidence (https://www.covidence.org/), duplicates were removed, and two reviewers (H.T.F. and M.B.) independently screened all titles and abstracts against predefined eligibility criteria (Table 1). Discrepancies were resolved by consensus. The PRISMA flow diagram is shown in Figure 1.

**TABLE 1:** Inclusion and exclusions criteria for study screening.

Inclusion criteria	Exclusion criteria
Stroke populations (ischemic or hemorrhagic) Blood‐derived biomarkers of NfL, GFAP, Aβ, tau, and PlGF (whole blood, plasma, and serum) measured after stroke Cognition assessed at ≥1 timepoint	Animal or non‐stroke studies (including TIAs) Incomplete texts, reviews, editorials, letters, perspectives, book chapters, retracted studies, conference papers, and errata Non–English‐language publications

Abbreviations: Aβ, amyloid beta; GFAP, glial fibrillary acidic protein; NfL, neurofilament light; PlGF, placental growth factor; TIA, transient ischemic attack.

### 2.2 Data extraction

Three reviewers (H.T.F., O.B., and M.B.) independently extracted data using a predefined template (Supplementary Material 2). Extracted variables included: (1) publication details, (2) study design and study period, (3) biomarker‐related data and analytical methods, (4) cognitive outcomes and definitions of PSCI, (5) group‐level covariates (age, sample size, sex, and education), and (6) clinical or functional outcomes.

### 2.3 Outcome measures

The primary outcome was post‐stroke cognition, assessed using global cognitive screening tools (e.g., Montreal Cognitive Assessment [MoCA], Mini‐Mental State Examination [MMSE], and the Telephone Interview for Cognitive Status [TICS‐40]), functional cognition (Cognitive Functional Independence Measure [FIM] subscale), detailed neuropsychological batteries, domain‐specific cognitive tasks, or clinical diagnoses of PSCI or dementia. For clarity, the term “cognition” is used throughout this review to refer to global cognitive performance on the MoCA, MMSE, or TICS‐40, unless otherwise specified; where studies examined domain‐specific cognitive functions or functional cognition, this is explicitly stated. Effect measures included correlation coefficients (Pearson's r, Spearman's rho), regression coefficients, and odds ratios (ORs). No restrictions were applied to biomarker measurement methods or timepoints post‐stroke. For synthesis, timepoints were pragmatically categorized as: acute (<1‐month post‐stroke), subacute (1–6 months), and chronic (>6 months) adapted from the framework of Bernhardt and colleagues.^40^

### 2.4 Risk of bias assessment

Risk of bias was assessed using a customized template based on the Cochrane Risk of Bias in Non‐Randomised Studies of Exposure (ROBINS‐E) tool.^41^ Domains evaluated included: (1) confounding factors, (2) participant inclusion criteria, (3) BBM measurement methodology, (4) missing data, (5) measurement of the cognitive outcome, and (6) selection of reported results. Overall study risk was determined by the highest‐risk domain.^41^ Assessments were performed independently by three reviewers (H.T.F., O.B., and M.B.), with disagreements resolved through discussion. Risk of bias visualizations were produced using the robvis tool.^42^

### 2.5 Data synthesis

Findings were synthesized narratively using a structured framework for each biomarker. Group‐level reporting was described, with cross‐sectional and longitudinal outcomes presented separately. Associations with cognition were summarized, covariate adjustments noted where available, and null findings were explicitly reported.

### 2.6 Statistical analysis

Meta‐analyses were conducted when ≥4 comparable studies were available for a given biomarker–cognition timepoint pairing (e.g., acute biomarker measurement vs chronic cognitive outcome). Pairings with <4 studies were synthesized qualitatively to avoid under‐powered analyses. When multiple effect measures were available for the same biomarker–cognition phase pairing, the most appropriately adjusted model was prioritized for reporting (accounting for relevant covariates without over‐adjustment). If no adjusted estimate was reported, the corresponding unadjusted value was extracted. Correlation coefficients were transformed to Fisher's Z for running the meta‐analysis but are presented as raw coefficients in text and figures. Analyses were conducted in R (v4.5.1) using the metafor package, applying random‐effects models (REMLs). Heterogeneity was assessed using τ^2^, I^2^ (with 95% confidence interval [CI]), and Cochran's Q statistic. Given the limited number of studies, small‐study effects (funnel plots, Egger's tests) were not assessed, as these are underpowered with <10 studies.^43^ Statistical significance was set at *p* < 0.05.

## 3 RESULTS

From 1746 records screened, 18 studies met inclusion criteria (Figure 1). These comprised 14 cohort studies, 3 cross‐sectional studies, and 1 randomized trial. Fourteen studies focused on ischemic stroke, three included mixed stroke types, and one examined hemorrhagic stroke. Sample sizes ranged from 24 to 1694 (interquartile range [IQR]: 67–245), with mean participant ages between 58.8 and 75.1 years. The exclusion criteria for many studies included individuals with pre‐existing cognitive impairment, major neurodegenerative or psychiatric disease, large or strategically located infarcts, severe systemic illness, or sensory or motor deficits precluding cognitive testing (Supplementary Material 3). Baseline medical histories and vascular co‐morbidities were reported variably across included studies (Supplementary Material 3). Furthermore, substantial heterogeneity was observed in pre‐analytical processing and biomarker sampling methods (Supplementary Materials 3 and 4) alongside cognitive outcome measures that spanned brief global screening instruments to detailed neuropsychological batteries (Table 2).

**TABLE 2:** Characteristics of screened studies.

Study [location]	Study type/population	Sample size, *n* (% male)	Age	Blood biomarker(s) measured	Blood sampling, days since stroke	Cognitive assessment, days since stroke	Cognitive outcome measure	PSCI definition
Chen et al. (2018) [China]^55^	L/M	30 (56.67%)	65.9 ± 8.7	Aβ40	On admission	On admission	MMSE and MoCA	MoCA <26 or MMSE <27
Chi et al. (2019) [Taiwan]^58^	L/IS	55 (81.8%)	59.8 ± 8.9	Aβ40, Aβ42; total tau	1, 90	90, 365	MoCA	MoCA <23
Egle et al. (2021) [UK]^47^	L/IS	113 (65.5%); 90 at 5 years	70.0 ± 9.9	NfL	90	90, then annually up to 5 years	Comprehensive Neuropsychological battery	N/A
Ferrari et al. (2023) [Italy]^44^	L/IS	36 (44.4%)	75.1 ± 11.8	GFAP; NfL	1, 7, 30, 90	1, 7, 30, 90	Cognitive‐FIM subscale	N/A
Gendron et al. (2020) [USA]^46^	L/M	314 (50.6%)	66.0 (18.8–100.7)	NfL	0–20	1	MMSE	N/A
Huang et al. (2021) [Taiwan]^57^	X/IS	24 (83.3%)	62.0 ± 8.5	Aβ40, Aβ42; total tau	90	90	CDR; DRS‐2; MMSE	N/A
Huang et al. (2022) [Taiwan]^60^	L / IS	136 (71.3%)	58.8 ± 12.7	Aβ40, Aβ42; total tau; p‐tau181	within 7 days	90, 365	CDR; MoCA (Taiwanese version)	CDR‐SB >0
Jiang et al. (2022) [China]^50^	L / IS	264 (59.5%)	65.0 ± 22	NfL	2	90	MoCA (Beijing edition)	MoCA <26
Li et al. (2025) [China]^54^	RCT / IS	622 (70.1%)	60.3 ± 11.4	NfL	1	90	MMSE	MMSE <27
Mao et al. (2020) [China]^61^	L / IS	188 (62.2%)	68.1 ± 10.4	Aβ42	1	7, 90, 180, 365	MoCA	MoCA <26
Peng et al. (2021) [USA]^45^	L / IS	144 (63.9%)	69.2 ± 10.9	NfL	7, 21	Admission; discharge (≤30 days)	Cognitive‐FIM subscale	N/A
Sanchez et al. (2024) [Canada]^48^	L / IS	161 (68.3%)	69.2 ± 7.4	Aβ40, Aβ42; GFAP; NfL; p‐tau181	90	90, 365, 730	Comprehensive Neuropsychological battery	N/A
Shi et al. (2024) [China]^56^	X / IS	83 (61.4%)	71.0 ± 10.0	Aβ42	within 3 days	within 3 days	MMSE	MMSE ≤26
Stokowska et al. (2021) [Sweden]^49^	L / M	115 (55.65%)	64.3 ± 6.42	NfL	10 months–5 years	10 months–5 years	Letter‐number sequence	N/A
Tang et al. (2018) [Taiwan]^59^	X / IS	61 (52.5%)	74.5 ± 6.96	Aβ40, Aβ42; total tau	71.2 ± 70.3 months	71.2 ± 70.3 months	MMSE; MoCA	N/A
Wang et al. (2021a) [China]^51^	L / IS	1694 (52.72%)	64.0 ± 16	NfL	2	90	MoCA	MoCA < 26
Wang et al. (2021b) [China]^53^	L / IS	304 (57.24%)	64.9 ± 9.2	NfL	30	395	TICS‐40 (Chinese version)	TICS‐40 ≤ 20 = MCI; ≤ 12 = dementia
Zheng et al. (2023) [China]^52^	L / H	26 (84.62%)	59.0 (27–84)	NfL	within 3 days; 7; 14	180	MoCA	N/A

Note. Age values are represented as either mean ± SD or median (IQR or range). Blood sampling and cognitive assessment times were represented as days since stroke unless otherwise stated. Sample size (*n*) refers to the number of unique human participants included in each study. Abbreviations: Aβ, amyloid beta; CDR‐SB, Clinical Dementia Rating– Sum of boxes; Cognitive‐FIM, Cognitive Functional Independence Measure; DRS‐2, Dementia Rating Scale 2; H, hemorrhagic; IS, ischemic stroke; L, Longitudinal; M, mixed; MMSE, Mini‐Mental State Examination; MoCA, Montreal Cognitive Assessment; NfL, neurofilament light; p‐tau, phosphorylated tau; PSCI, post‐stroke cognitive impairment ; RCT, randomised controlled trial; TICS‐40, Telephone Interview for Cognitive Status 40; X, cross‐sectional.

### 3.1 NfL

NfL–cognition associations were examined in 11 studies; see Supplementary Material 4 for cohort characteristics.^44^, ^45^, ^46^, ^47^, ^48^, ^49^, ^50^, ^51^, ^52^, ^53^, ^54^

#### 3.1.1 Cross‐sectional

Higher acute NfL concentrations were consistently associated with poorer functional cognition at 7 days (rho = –0.84, *p* < 0.001)^44^ and 11 days (rho = –0.39, *p* < 0.05),^45^ and cognition 20 days post‐stroke (OR = 1.83, 95% CI: 1.55–2.20, adjusted for clinical covariates).^46^ Subacute associations were modest, with increased NfL at 30–90 days linked to worse functional cognition (rho = ‐0.53 to ‐0.63, *p* < 0.05)^44^ and global cognitive function, executive function, and processing speed (*β* = –0.15 to –0.24, *p* < 0.05),^47^ although only the latter remained significant after adjustment for neuroimaging and clinical covariates (*β* = –0.187, *p* < 0.05). Other studies reported no significant subacute associations.^48^ In one chronic‐phase study, higher NfL modestly associated with poorer working memory, after adjustment for age and stroke severity (rho = –0.21, *p* < 0.05; *β* range −1.23 to −1.59, *p* < 0.05).^49^

#### 3.1.2 Longitudinal

Five studies demonstrated that higher acute NfL concentrations predicted poorer subacute cognition.^44^, ^45^, ^50^, ^51^, ^52^ Higher acute NfL predicted poorer functional cognition at (1) 30 days (*β* = –0.321, *p* < 0.001; adjusted for baseline function [mRS], infarct, and SVD volumes),^45^ and (2) 90 days (*β* = –0.002, *p* < 0.001; adjusted for age, mRS, and Fazekas SVD score).^44^ In the latter, the association was no longer significant when GFAP concentrations were included as an additional covariate, suggesting potential confounding or mediation by GFAP in the early post‐stroke phase.

**FIGURE 2:**
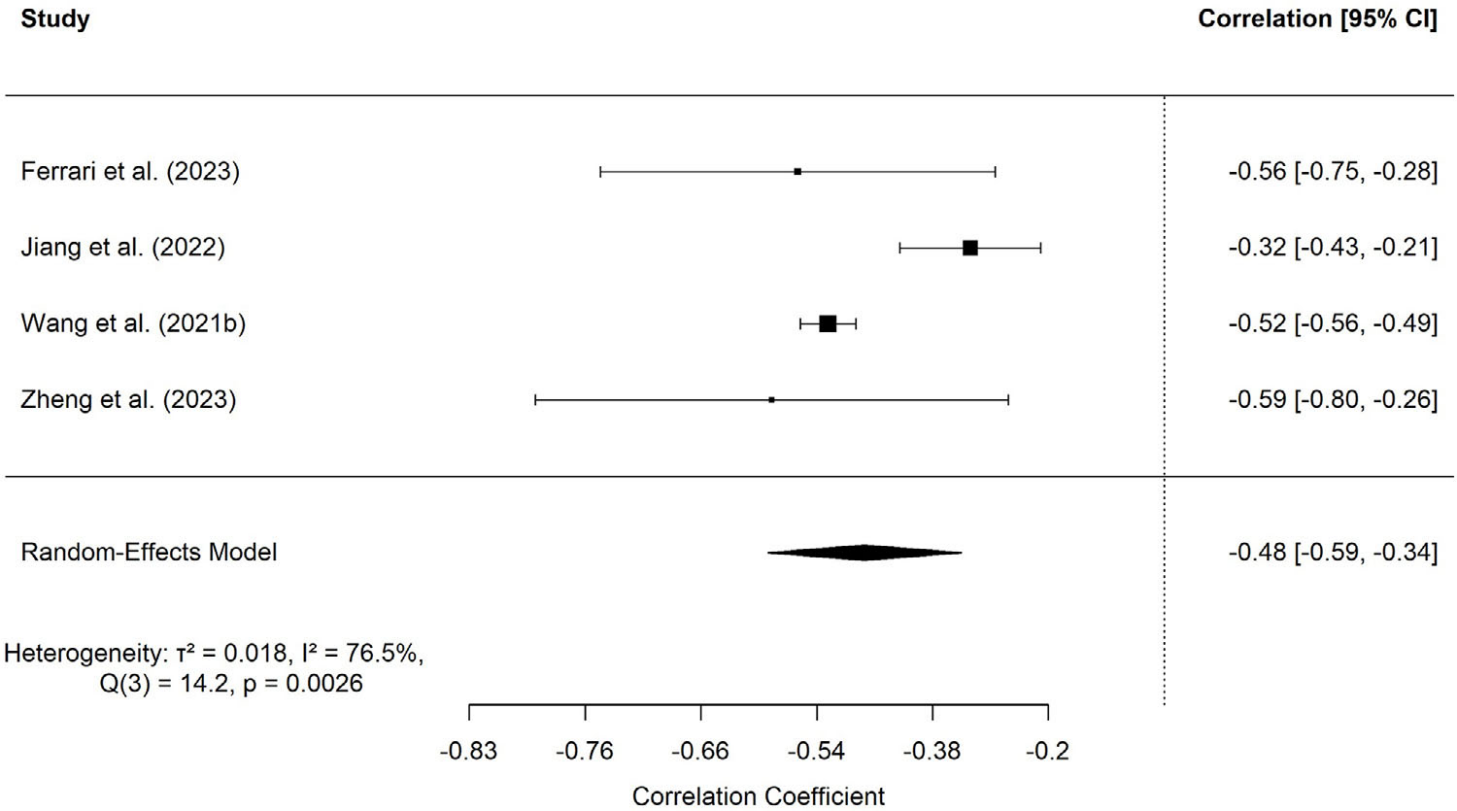
Random‐effects meta‐analysis of the association between acute blood neurofilament light (NfL) (<1‐month post‐stroke) and subacute cognitive outcomes (1–6 months post‐stroke).

Larger well‐adjusted cohort studies confirmed the longitudinal predictive value of acute‐phase NfL (OR = 1.04 per log10 unit increase in NfL, all results *p* < 0.001; adjusted for age, gender, education level, baseline stroke severity and imaging covariates),^50^, ^51^ including in haemorrhagic stroke (rho = ‐0.59, *p* < 0.001).^52^ A random‐effects meta‐analysis of four studies (*n* = 2020) demonstrated a moderate‐to‐strong association between higher acute NfL and poorer subacute cognition (Fisher *Z* = –0.518, SE = 0.085, *p* < 0.0001; 95% CI: −0.684 to −0.351) (Figure 2).^44^, ^50^, ^51^, ^52^ Substantial heterogeneity was present (*τ^2^* = 0.018, *I^2^* = 76.5%, Q(3) = 14.24, *p* = 0.0026), consistent with between‐study differences in participant characteristics, stroke subtypes, and the timing of biomarker and cognitive assessments. Subacute NfL levels also predicted chronic cognitive outcomes.^47^, ^53^ At 30 days, higher NfL was associated with poorer cognition at 1 year (OR = 1.06–1.07, *p* < 0.001) and reduced cognitive improvement over time (*β* = –0.28 to –0.31, p < 0.001).^53^ Similarly, higher NfL levels at 90 days predicted worse global cognition at 5 years (*β* = –0.30 to –0.34, *p* < 0.002) and increased dementia risk (hazard ratio [HR] = 1.45–1.68, all *p* < 0.05 for different adjusted models).^47^ Finally, NfL measured in the chronic phase after stroke was not associated with letter–number sequence performance over a 9‐month follow‐up period (OR = 1.47, 95% CI: 0.88–2.53, *p* = 0.15).^49^

Beyond its prognostic value, NfL measured within 24 h of admission may also moderate treatment response; Li and colleagues^54^ found that early antihypertensive therapy initiated in the acute phase reduced PSCI risk at 3‐month follow‐up in patients with low baseline NfL (OR = 0.50, 95% CI: 0.31–0.81) but increased it in those with high baseline NfL (OR = 1.93, 95% CI: 1.16–3.20), with a significant interaction between NfL level and treatment (*p* < 0.001). However, as this clinical trial enrolled a younger‐than‐typical stroke population, this finding requires replication in prospective studies before clinical translation.

### 3.2 Amyloid

Eight studies examined amyloid species in relation to cognition, providing both cross‐sectional and longitudinal analyses; see Supplementary Material 4 for cohort characteristics.^48^, ^55^, ^56^, ^57^, ^58^, ^59^, ^60^, ^61^

#### 3.2.1 Cross‐sectional

In the acute phase, higher Aβ1‐40 concentrations were associated with worse cognition (r = –0.874, *p* < 0.01, unadjusted)^55^ and higher Aβ1‐42 was associated with increased odds of cognitive impairment (OR = 1.009, 95% CI: 1.00–1.018, *p* = 0.045; unadjusted).^56^ Findings were more heterogeneous in the subacute phase. Higher Aβ1‐40 was associated with better cognitive performance (*r* = 0.38–0.44, *p* < 0.05), whereas higher Aβ1–42/40 ratio was associated with poorer cognition (*r* = −0.32 to −0.44, *p* < 0.05); associations with Aβ1‐42 alone were non‐significant. These patterns persisted after adjustment for age and education.^57^ However, larger studies did not replicate these findings (OR = 0.88–1.04, all *p*’s > 0.25).^48^, ^58^ In the chronic phase, a single paper reported no association with Aβ1‐40 levels and cognition (*r* = 0.07–0.17, *p* > 0.25) but found that higher Aβ1‐42 was associated with poorer cognition (*r* = –0.44 to –0.46, *p* < 0.01).^59^

#### 3.2.2 Longitudinal

Across three studies examining longitudinal associations between plasma amyloid and cognition, the evidence was mixed.^58^, ^60^, ^61^ Two studies found no evidence that acute amyloid concentrations or ratios were associated with cognition at subacute or chronic follow‐up (*p* > 0.1).^58^, ^60^ In contrast, a larger cohort study by Mao and colleagues (2020), which assessed Aβ1‐42 concentrations only, reported that higher acute Aβ1‐42 was associated with better cognition (rho = 0.348, *p* < 0.05) and a lower risk of cognitive impairment at 1 year (relative risk = 0.282, 95% CI: 0.258–0.833, *p* < 0.05; adjusted for demographic, vascular, and clinical variables).^61^ Finally, higher subacute Aβ1‐42 was associated with improved cognition at 1 year (OR = 0.63, *p* < 0.05; adjusted for baseline cognitive scores), whereas the Aβ1‐42/40 ratio showed a similar but weaker, borderline association (OR = 0.87, 95% CI: 0.76–1.00, *p* = 0.055).^58^

### 3.3 Tau

Five studies assessed plasma tau (t‐tau and phosphorylated) concentrations using cross‐sectional and longitudinal designs; see Supplementary Material 4 for cohort characteristics.^48^, ^57^, ^58^, ^59^, ^60^

Cross‐sectionally, higher subacute plasma t‐tau showed weak‐to‐moderate associations with poorer cognition (r = −0.27 to −0.46, *p* < 0.05; adjusted for age, education, and depression).^57^ Other subacute and chronic cross‐sectional studies reported no significant associations between t‐tau and cognition (*p* > 0.05).^58^, ^59^ Longitudinally, acute t‐tau concentrations did not predict cognitive outcomes at subacute or chronic follow‐up.^58^, ^60^ However, one study reported that higher subacute tau concentrations were associated with a lower risk of PSCI at 1 year (OR = 0.85, 95% CI: 0.73–0.99; adjusted for education), a finding that contrasts with the cross‐sectional associations and should therefore be interpreted cautiously.^58^

Two studies examined p‐tau181, yielding inconsistent and potentially counter‐intuitive findings.^48^, ^60^ Huang and colleagues (2022) reported that higher acute p‐tau181 was associated with a reduced risk of PSCI subacutely (OR = 0.62, 95% CI: 0.40–0.94) and chronically (OR = 0.69, 95% CI: 0.47–0.99).^60^ Conversely, Sanchez and colleagues (2024) reported a borderline association between higher log10p‐tau181 and poorer cognition that did not reach statistical significance (*B* = –0.32, *p* = 0.062).^48^ Several differences may underlie these conflicting results. Notably, the Huang cohort was approximately a decade younger than that of Sanchez et al. (2024),^48^ potentially reducing the prevalence of underlying Alzheimer's‐type pathology. In addition, p‐tau181 was quantified using different assay platforms (Immunomagnetic Reduction [IMR] vs Single Molecular Array [SIMOA]), and cognitive outcomes were assessed using a screening instrument (MoCA) rather than a comprehensive neuropsychological battery, which may have limited sensitivity to post‐stroke cognitive impairment. Although Huang et al. adjusted for several vascular and clinical covariates, key imaging markers such as infarct volume were not included. Taken together, differences in population characteristics, biomarker measurement, and cognitive assessment, combined with small sample sizes, limit confidence in the direction and interpretability of p‐tau181 associations with post‐stroke cognition.

### 3.4 GFAP

Only two studies investigated associations between GFAP concentrations and cognition (see Supplementary Material 4 for cohort characteristics).^44^, ^48^ Both studies reported cross‐sectional associations in the subacute phase; higher GFAP was associated with poorer functional cognition and worse attention, working memory, and executive function (rho = –0.63, *p* < 0.001); (*B* = −0.42 to −0.45, *p* < 0.05; adjusted for age, sex, education, and apolipoprotein E [*APOE*] ε4 status).^44,^ ^48^ Ferrari and colleagues (2023) additionally observed strong negative correlations between acute‐phase GFAP concentrations and functional cognition (rho = –0.77, *p* < 0.0001). Of note, this study examined baseline GFAP concentrations in relation to subsequent cognitive outcomes, with higher GFAP associated with poorer subacute functional cognitive outcomes (rho = –0.73 and –0.64; both *p* < 0.01) and predicted worse cognition at 90 days (*β* = –0.009, *p* < 0.05; adjusted for age, stroke severity, and SVD burden). However, this association was no longer significant after additional adjustment for NfL, suggesting that GFAP‐related effects may overlap with, or be mediated by, neuroaxonal injury.^44^

### 3.5 Risk of bias assessment

**FIGURE 3:**
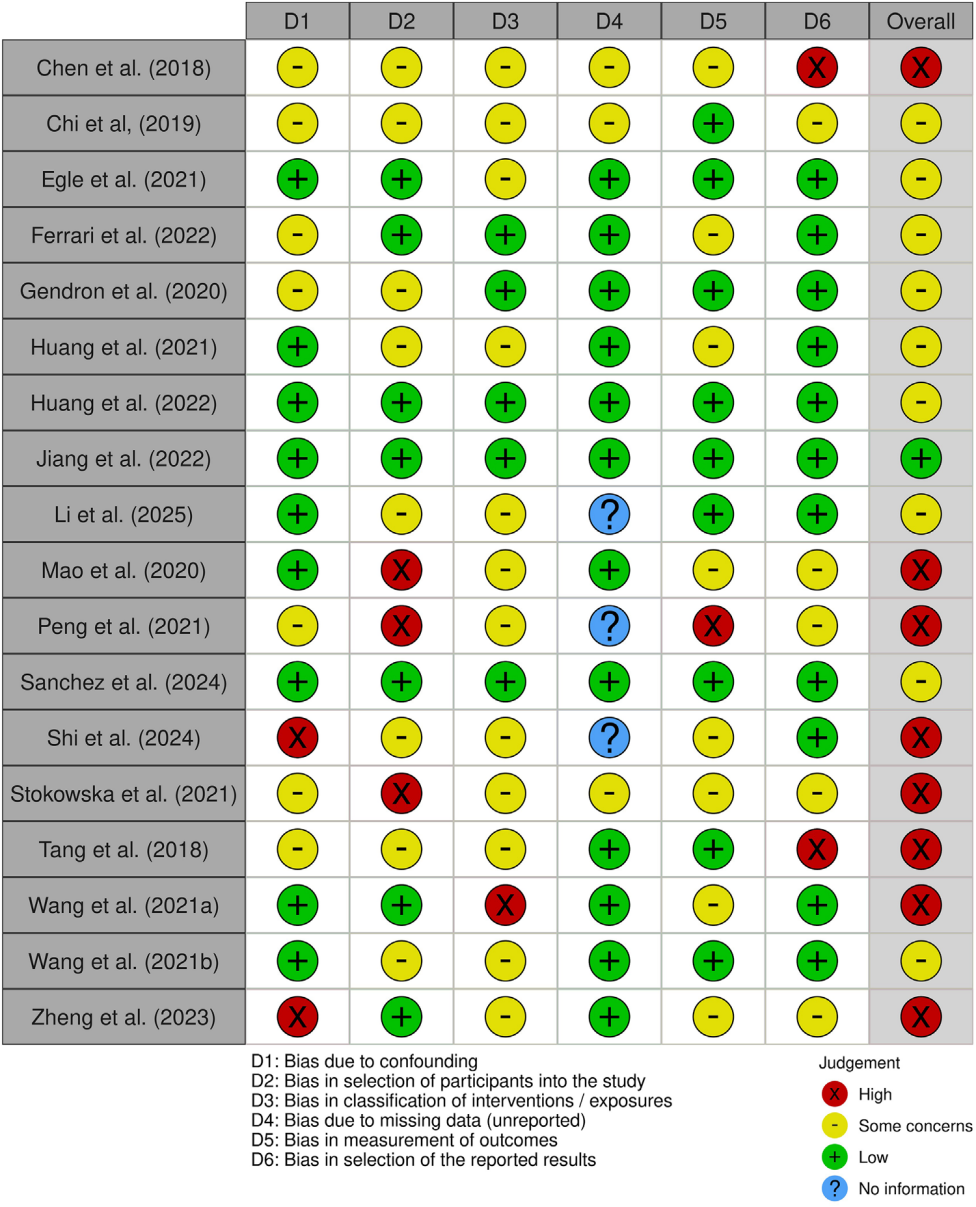
Risk of bias assessment was conducted using an adaptation of the Risk of Bias in Non‐Randomised Studies of Exposure (ROBINS‐E) tool^41^. The overall risk of bias was based on the highest risk domain ^41^. Visualization of the risk of bias assessments were performed with the Risk‐of‐bias VISualisation (robvis) tool ^42^.

Eight of 18 studies were judged to be at high overall risk of bias, most frequently due to concerns in participant selection and selective reporting of results (Figure 3). Within each bias domain, between 0–3 studies were judged to have high risk of bias; the domain most often rated as high risk was participant selection (three studies). The justification for the full domain‐level assessments for each study is available in Supplementary Materials 5.

## 4 DISCUSSION

This systematic review synthesised evidence on dementia‐related BBMs (NfL, GFAP, Aβ and tau species, and PlGF) in relation to post‐stroke cognitive outcomes. Eighteen studies met inclusion criteria, most focusing on NfL (*n* = 11), followed by amyloid‐ and tau‐related biomarkers and GFAP. Across studies, NfL and GFAP showed the most consistent associations with PSCI, whereas evidence for amyloid‐ and tau‐related biomarkers was more heterogeneous between studies and suggested weaker and less reliable predictive utility. Although PlGF has been linked to cognitive outcomes in cerebral SVD cohorts,^19^ no eligible studies examined PlGF in clinical stroke populations within the review window. Notably, few studies were judged to have a low risk of bias across all domains, highlighting the need for more rigorous study designs, better‐controlled cohorts, and standardized biomarker and cognitive assessment protocols.

NfL is an established marker of neuroaxonal injury and is consistently linked to vascular neuroimaging abnormalities, functional outcomes after stroke, and neurodegenerative disease including AD.^9^, ^26^, ^27^, ^28^ The meta‐analysis revealed that acute‐phase NfL concentrations had the strongest associations with later cognitive performance, particularly during the subacute period (1–6 months). When measured acutely (<1 month), NfL concentrations primarily reflect the extent of initial neuroaxonal injury from the index stroke, before inflammatory, neuroplastic, and recovery‐related processes introduce additional sources of variance. Acute stroke produces axonal damage, and early NfL concentrations likely reflect the scale of this injury, including tissue lost through secondary degeneration such as Wallerian degeneration, which may contribute to the characteristic post‐stroke NfL peak observed at ≈1–3 months.^29^, ^30^

During the subacute phase, cognitive performance most directly reflects this initial injury burden, before longer‐term factors such as neural plasticity, recovery trajectories, small vessel disease, and ongoing neurodegeneration increasingly shape cognitive outcomes. Although NfL concentrations continue to rise toward their biological peak during this period, cognitive performance often improves as compensatory mechanisms begin to offset the effects of the initial insult. As these processes diverge over time, associations between NfL and cognition are expected to attenuate. Overall, these findings suggest that NfL is most informative when measured acutely after stroke as a marker of initial neuroaxonal injury burden, rather than as a standalone indicator of longer‐term cognitive recovery.

Across the included studies, associations between amyloid and tau species and post‐stroke cognition were highly heterogeneous, with effect sizes and directions varying across cross‐sectional and longitudinal designs. This heterogeneity contrasts with non‐stroke dementia cohorts, where associations between these biomarkers and cognitive decline are more consistent.^14^, ^62^, ^63^, ^64^ In this review, it remains unclear whether amyloid and tau reflect parallel AD‐related pathological processes associated with post‐stroke cognitive outcomes or simply act as bystanders in post‐stroke trajectories. Interpretation was hampered by methodological limitations, particularly the inadequate adjustment for well‐established post‐stroke cognitive predictors such as stroke volume, lesion characteristics, and topology. Most participants were 60–70 years of age, an age range at which significant Alzheimer's‐related cognitive sequelae may not yet have emerged, perhaps reducing statistical power to detect associations. The following sections consider amyloid and tau separately, discussing potential reasons underlying these biomarker‐specific patterns.

Traditionally, lower Aβ42 levels in non‐stroke dementia cohorts are associated with worse cognitive trajectories, reflecting sequestration into plaques in AD.^62^, ^63^, ^65^ In this review, associations between Aβ and post‐stroke cognition differed from those typically observed in non‐stroke dementia cohorts. Specifically, for Aβ42, some studies reported that higher Aβ42 was associated with worse cognition across all timepoints cross‐sectionally. Worse outcomes following increased Aβ42 concentrations have also been reported across stroke studies examining other outcomes. For example, in spontaneous hemorrhage, higher admission Aβ42 predicted worse 3‐month functional outcomes (mRS ≥2) despite no association with age or stroke severity.^24^ Two mechanisms may explain these divergent patterns observed in stroke settings: (1) acute vascular injury may perturb pre‐existing amyloid plaques, transiently elevating circulating Aβ42, as observed in amyloid precursor protein/presenilin 1 (APP/PS1)  mouse work investigating microvascular occlusion,^66^ and (2) cognition in the acute period may be driven by stroke‐related factors, leaving little variance for Aβ42 to explain cognitive performance. This aligns with evidence that elevated Aβ42 relates more strongly to cognition before incident stroke.^67^ Of interest, some longitudinal studies from this review aligned with traditional amyloid biology, with higher Aβ42 and Aβ42/40 ratios predicting better long‐term outcomes.^58^, ^61^ Taken together, Aβ42 may be more informative as a predictor of longer‐term recovery than as an index of acute cognitive status or injury burden, although evidence remains sparse and hypotheses require validation in larger clinical cohorts.

Evidence for an association between t‐tau and post‐stroke cognition was inconsistent, with weak cross‐sectional associations observed in the subacute phase but no reliable longitudinal predictive signal. Findings for p‐tau181 were similarly inconsistent, with higher concentrations associated with both reduced risk of PSCI and poorer cognitive performance across studies, although these effects differed in statistical strength and methodological adjustment.^48^, ^60^ One explanation for this heterogeneity is that only 20% of circulating tau originates from the central nervous system (CNS),^68^ limiting the specificity of tau as a marker for stroke‐related neuroaxonal injury. Brain‐derived tau (or BD‐tau) may overcome this limitation, as it exhibits greater CNS correlations compared to the non–brain‐derived counterparts,^69^ and has demonstrated prognostic relevance in vascular injury; BD‐tau was associated with poorer 90‐day functional outcomes (mRS ≥3),^25^ correlated with cortical stroke diameter, and differentiates acute ischemic stroke from mimic presentations.^70^ However, whether tau changes contribute to post‐stroke cognitive outcomes remains unclear, and exploration of AD‐related phosphoforms (p‐tau181, p‐tau217), and their emerging brain‐derived counterparts, remain underexplored in stroke populations.

From the limited number of studies included in this review (*n* = 2), findings suggest a negative association between GFAP and post‐stroke cognition. Emerging work (published after the search date) supports this pattern; elevated GFAP measured <7 days of the ictus predicted PSCI at 3 months independent of age and sex (OR = 3.91, 95% CI: 2.24–6.82; MMSE <27).^71^ GFAP, expressed almost exclusively in astrocytes, rises rapidly after stroke and has been linked to stroke severity and poorer outcomes.^31^, ^32^ This review provides preliminary support for an association between GFAP and cognitive trajectories following stroke. However, whether GFAP adds predictive value beyond other biomarkers or imaging remains unclear. Notably, no studies measured GFAP longitudinally across multiple post‐stroke timepoints in relation to cognition, thereby limiting conclusions about how its temporal trajectory relates to cognitive outcomes. Moreover, neither study within this review included hemorrhagic populations. Therefore, the potential role of GFAP as a cognitive BBM in haemorrhagic stroke is unexplored.

No studies in this review assessed PlGF in relation to PSCI, likely because it has only recently emerged as a potential BBM for vascular contributions to cognitive impairment in the U.S.‐based MarkVCID study.^19^, ^72^ Notably, a study published beyond the search period reported higher PlGF levels to be associated with poorer cognition after acute lacunar or minor cortical stroke, after adjustment for key demographic variables and baseline SVD burden.^73^ PlGF, part of the vascular endothelial growth factor family, promotes arteriogenesis and angiogenesis.^74^, ^75^, ^76^ Circulating PlGF rises during ischemia and may reflect vascular stress and risk factor burden.^34^ However, findings from other studies that investigate non‐cognitive post‐stroke outcomes remain inconsistent, some studies reporting no association,^77^ and others linked higher PlGF to increased risk of cardiovascular events.^34^ PlGF may therefore act as both a marker of systemic vascular stress and a mediator of repair, but its relevance to post‐stroke cognitive outcomes requires further evaluation.

### 4.1 Limitations of the evidence included in the review

A key limitation of this review is the small number of eligible studies and, more specifically, the limited and uneven evidence base for each biomarker at the time of the literature search. This was particularly evident in the absence of studies examining other phosphorylated tau phosphoforms, including p‐tau217 and p‐tau231, as well as PlGF. With so few data points, heterogeneity in study design, cohort characteristics, and analytical approaches exert a disproportionate influence on overall conclusions.

Substantial variability in blood sampling windows after stroke further prevented pooling of findings in a formal meta‐analysis, limiting the precision and robustness of synthesised estimates. Moreover, 8 of the 18 included studies were judged to be at high risk of bias, most often due to insufficient participant selection reporting, which may have influenced their reported outcomes.

Sample sizes were relatively small in many studies. Although some smaller cohorts reported comparatively large associations, findings from larger, better‐controlled studies suggested more moderate but likely more reliable effect sizes, particularly for NfL. Moreover, only four studies included patients with hemorrhagic stroke, making it difficult to draw the same conclusions to non‐ischemic populations. Studies specifically examining biomarker trajectories and cognitive outcomes in hemorrhagic stroke populations are therefore required.

Another major limitation was inconsistent and often incomplete adjustment for key covariates when modeling cognitive outcomes after stroke. Imaging metrics such as stroke volumes or lesion locations, prior stroke history, important co‐morbidities such as renal impairment, and functional outcomes such as the mRS and NIHSS were often not adjusted for despite evidence supporting their associations with PSCI.^6^, ^78^, ^79^, ^80^ Moreover, even when clinical and imaging markers of CVD were available, they were not consistently incorporated into statistical models, reflecting absent or heterogeneous data collection and reporting. In studies presenting both adjusted and unadjusted estimates, associations commonly attenuated or lost their statistical significance once adjustments were applied (e.g., Egle and colleagues),^47^ suggesting that many of the reported findings may lack clinical validity due to unaccounted confounders.

Methodological heterogeneity extended beyond covariate adjustment to include substantial variability in pre‐analytical and analytical procedures. Pre‐analytical factors such as centrifugation protocols, processing time, and storage conditions differed widely across studies and were reported inconsistently. These have been reported to impact biomarker concentration measurements.^81^ Furthermore, the use of different assay platforms, particularly for Aβ quantification, introduces more variability, as assay performance depends on antibody epitope specificity, which may differentially capture monomeric versus oligomeric species.^82^

Finally, cognitive assessments imposed further constraints on interpretability. Most relied on brief global screening tools such as the MoCA or MMSE. Although widely used, these tools were developed for dementia screening rather than post‐stroke populations^83^, ^84^ and do not capture domain‐specific deficits frequently observed after focal vascular injury, such as aphasia, neglect, apraxia, dyslexia, executive dysfunction, or visuospatial impairment.^5^, ^85^, ^86^ Notably, PSCI was variably defined when using these tools (Table 2). Moreover, many of the included studies were conducted in East Asian populations, where these instruments were not originally normed. Although some studies used culturally adapted versions or adjusted for education, this was inconsistent across studies, potentially affecting cutoff validity and cross‐study comparability. Only a minority of studies employed comprehensive neuropsychological batteries, limiting characterisation of subtle or heterogeneous PSCI profiles. Reliance on coarse measures raises the risk of misclassification or underestimation and reduces comparability between studies due to ceiling effects and variable sensitivity.

### 4.2 Limitations of the review process

To facilitate synthesis and reporting, outcomes were grouped into acute (<1‐month post‐stroke), subacute (1–6 months), and chronic (>6 months) timepoints. These categories differ slightly from the consensus framework of Bernhardt and colleagues (acute = 0–7 days; subacute = 7 days to 6 months; chronic = >6 months)^40^ but were pragmatically selected to accommodate heterogeneous sampling schedules and enable pooling for potential meta‐analysis. Nevertheless, blood protein concentrations may fluctuate markedly within these broad intervals. For example, the hyper‐acute phase (<24 hours) is characterized by variations in NfL and GFAP levels.^29^, ^31^ By grouping studies into wider bands, associations specific to this early window may have been obscured. However, the limited number of studies prevented finer stratification. Pooled estimates and heterogeneity measures should therefore be interpreted with caution.

Another limitation relates to the scope of BBMs included. This review deliberately focused on dementia‐related BBMs, meaning other biological pathways relevant to post‐stroke recovery were not examined. Consequently, associations involving inflammatory markers (e.g., C‐reactive protein [CRP], interleukin 6 [IL‐6], tumor necrosis factor α [TNFα]) or metabolic and vascular indicators (e.g., homocysteine, cholesterol, low‐density lipoprotein [LDL], uric acid) were not investigated. These pathways have been explored in previous reviews,^87^, ^88^ and the intention was not to replicate this work but to address a specific evidence gap concerning emerging dementia‐related BBMs and post‐stroke cognition.

Finally, risk of bias assessments are inherently subjective. Although the rationale for each judgement is outlined in Supplementary Materials 5, alternative interpretations are possible. Conducting assessments independently by three reviewers before consensus discussions helped enhance rigour and reliability.

### 4.3 Implications for future research

There remains an unmet need for vascular‐specific BBMs that can reliably predict vascular cognitive impairment.^4^ Well‐controlled studies are required to clarify the added value of dementia‐related BBMs for longitudinal forecasting of post‐stroke cognitive outcomes. Scaling these investigations will likely require broader adoption of digital cognitive assessments, which enable high‐frequency, low‐burden, and large‐cohort data collection.^38^, ^86^

As highlighted throughout this review, many studies inadequately accounted for stroke‐specific factors that strongly influence BBM–cognition relationships. Future research should incorporate detailed neuroimaging‐derived measures of vascular injury such as lesion volume and topology, global SVD burden (WMHs and microbleeds),^89^ and markers of pre‐existing atrophy, all established predictors of PSCI.^78^, ^79^ Advanced magnetic resonance imaging (MRI) measurements of diffusion, perfusion, and blood–brain barrier (BBB) breakdown may further refine mechanistic interpretations of biomarker–cognition associations.^90^, ^91^ Beyond dementia‐related BBMs, a broader range of molecular mechanisms may contribute to vascular cognitive impairment and PSCI. Hosoki and colleagues outlined six such pathways: (1) endothelial dysfunction, (2) blood‐brain barrier BBB breakdown, (3) oxidative stress, (4) inflammation, (5) clotting pathway dysfunction, and (6) neuronal and glial degeneration.^17^ Although BBMs exist for each domain, their relevance to post‐stroke cognition remains underexplored. Future studies may benefit from examining composite or multimodal panels that capture signals from multiple interconnected pathways (e.g., PlGF for endothelial dysfunction, metalloproteinases for BBB disruption, and IL‐6 for inflammation).

Finally, interpretation of BBM concentrations must consider physiological factors influencing protein expression and clearance, including renal function,^92^ hepatic function,^93^ and fasting status.^94^ Although these factors cannot always be fully controlled, future research should document and adjust for them wherever possible to improve accuracy and interpretability of biomarker–cognition relationships.

## 5 CONCLUSION

This systematic review identified NfL and GFAP as two of the most consistently associated dementia‐related BBMs with post‐stroke cognitive outcomes, particularly when measured in the acute phase. In contrast, evidence for amyloid‐ and tau‐related proteins was inconsistent or contradictory, suggesting limited prognostic value in the currently available study designs. No eligible studies examined PlGF in relation to post‐stroke cognitive outcomes within the review period, representing an important gap for future research. Interpretation of these findings is constrained by heterogeneity in study design, small sample sizes, and variable risk of bias. Large, well‐controlled longitudinal studies with standardized biomarker protocols, comprehensive cognitive assessments, and detailed neuroimaging data are needed to clarify the mechanistic and predictive relevance of these BBMs. Examining these biomarkers alongside other process‐specific BBMs for endothelial dysfunction, inflammation, and BBB disruption through multimodal biomarker panels, may further elucidate the biology of PSCI and the vascular contributions to dementia.

## CONFLICT OF INTEREST STATEMENT

Atticus H. Hainsworth has received honoraria from Eli‐Lilly and from National Institute on Aging (NIA). He serves as a consultant for AriBio Co. Ltd and chairs the Dementias Platform‐UK Vascular Experimental Medicine group. Paul M. Matthews is a consultant for Biogen, Nodthera, Sudo Therapeutics, GSK, and Novartis and has received research funding from Biogen, Merck, and Bristol Meyers Squibb. Henrik Zetterberg has served at scientific advisory boards and/or as a consultant for Abbvie, Acumen, Alector, Alzinova, ALZpath, Amylyx, Annexon, Apellis, Artery Therapeutics, AZTherapies, Cognito Therapeutics, CogRx, Denali, Eisai, Enigma, LabCorp, Merck Sharp & Dohme, Merry Life, Nervgen, Novo Nordisk, Optoceutics, Passage Bio, Pinteon Therapeutics, Prothena, Quanterix, Red Abbey Labs, reMYND, Roche, Samumed, ScandiBio Therapeutics AB, Siemens Healthineers, Triplet Therapeutics, and Wave; has given lectures sponsored by Alzecure, BioArctic, Biogen, Cellectricon, Fujirebio, LabCorp, Lilly, Novo Nordisk, Oy Medix Biochemica AB, Roche, and WebMD; is a co‐founder of Brain Biomarker Solutions in Gothenburg AB (BBS), which is a part of the GU Ventures Incubator Program; and is a shareholder of CERimmune Therapeutics (outside submitted work). Hing Tim Fung, Olivia Burton, Mara Bortnowschi, Laura M Parkes, and Fatemeh Geranmayeh declare no conflicts of interest. Author disclosures are available in the Supporting Information.

## Supporting information



Supporting Information

Supporting Information

Supporting Information

Supporting Information

Supporting Information

Supporting Information

## References

[1] Smith EE , Aparicio HJ , Gottesman RF , et al. Vascular contributions to cognitive impairment and dementia in the united states: prevalence and incidence: a scientific statement from the American Heart Association. Stroke. 2025;56(10):e317‐e330. doi:10.1161/STR.0000000000000494 40820756

[2] Iturria‐Medina Y , Sotero RC , Toussaint PJ , et al. Early role of vascular dysregulation on late‐onset Alzheimer's disease based on multifactorial data‐driven analysis. Nat Commun. 2016;7(1):11934. doi:10.1038/ncomms11934 27327500 PMC4919512

[3] Livingston G , Huntley J , Sommerlad A , et al. Dementia prevention, intervention, and care: 2020 report of the *Lancet* Commission. *The* Lancet. 2020;396(10248):413‐446. doi:10.1016/S0140-6736(20)30367-6 32738937 PMC7392084

[4] The VasCog‐2‐WSO Criteria Consortium , Sachdev PS , Bentvelzen AC , et al. Revised diagnostic criteria for vascular cognitive impairment and dementia—the VasCog‐2‐WSO criteria. JAMA Neurol. doi:10.1001/jamaneurol.2025.3242

[5] Demeyere N , Riddoch MJ , Slavkova ED , et al. Domain‐specific versus generalized cognitive screening in acute stroke. J Neurol. 2016;263(2):306‐315. doi:10.1007/s00415-015-7964-4 26588918 PMC4751179

[6] Pendlebury ST , Rothwell PM . Incidence and prevalence of dementia associated with transient ischaemic attack and stroke: analysis of the population‐based Oxford Vascular study. Lancet Neurol. 2019;18(3):248‐258. doi:10.1016/S1474-4422(18)30442-3 30784556 PMC6390174

[7] Feigin VL , Brainin M , Norrving Bo , et al. World stroke organization: global stroke fact sheet 2025. Int J Stroke. 2025;20(2):132‐144. doi:10.1177/17474930241308142 39635884 PMC11786524

[8] Frisoni GB , Hansson O , Nichols E , et al. New landscape of the diagnosis of Alzheimer's disease. Lancet. 2025;406(10510):1389‐1407. doi:10.1016/S0140-6736(25)01294-2 40997838

[9] Mattsson N , Andreasson U , Zetterberg H , Blennow K ; Alzheimer's disease neuroimaging initiative. association of plasma neurofilament light with neurodegeneration in patients with Alzheimer disease. JAMA Neurol. 2017;74(5):557‐566. doi:10.1001/jamaneurol.2016.6117 28346578 PMC5822204

[10] Shir D , Graff‐Radford J , Hofrenning EI , et al. Association of plasma glial fibrillary acidic protein (GFAP) with neuroimaging of Alzheimer's disease and vascular pathology. Alzheimer's Dement (Amst). 2022;14(1):e12291. doi:10.1002/dad2.12291 35252538 PMC8883441

[11] Keshavan A , Pannee J , Karikari TK , et al. Population‐based blood screening for preclinical Alzheimer's disease in a British birth cohort at age 70. Brain. 2021;144(2):434‐449. doi:10.1093/brain/awaa403 33479777 PMC7940173

[12] Li Y , Schindler SE , Bollinger JG , et al. Validation of Plasma Amyloid‐β 42/40 for Detecting Alzheimer Disease Amyloid Plaques. Neurology. 2022;98(7):e688‐e699. doi:10.1212/WNL.0000000000013211 34906975 PMC8865895

[13] Mattsson N , Zetterberg H , Janelidze S , et al. Plasma tau in Alzheimer disease. Neurology. 2016;87(17):1827‐1835. doi:10.1212/WNL.0000000000003246 27694257 PMC5089525

[14] Moscoso A , Grothe MJ , Ashton NJ , et al. Longitudinal associations of blood phosphorylated tau181 and neurofilament light chain with neurodegeneration in Alzheimer disease. JAMA Neurol. 2021;78(4):396‐406. doi:10.1001/jamaneurol.2020.4986 33427873 PMC7802009

[15] Mendes AJ , Ribaldi F , Lathuiliere A , et al. Head‐to‐head study of diagnostic accuracy of plasma and cerebrospinal fluid *p*‐tau217 versus *p*‐tau181 and *p*‐tau231 in a memory clinic cohort. J Neurol Published online 2024. doi:10.1007/s00415-023-12148-5 PMC10972950 38195896

[16] Chong JR , Chai YL , Yam ATY , et al. Association of plasma GFAP with elevated brain amyloid is dependent on severity of white matter lesions in an Asian cognitively impaired cohort. Alzheimer's Dement (Amst). 2024;16(2):e12576. doi:10.1002/dad2.12576 38605996 PMC11007806

[17] Hosoki S , Hansra GK , Jayasena T , et al. Molecular biomarkers for vascular cognitive impairment and dementia. Nat Rev Neurol. 2023;19(12):737‐753. doi:10.1038/s41582-023-00884-1 37957261

[18] Wu L‐Y , Chong JR , Chong JPC , et al. Serum placental growth factor as a marker of cerebrovascular disease burden in alzheimer's disease. J Alzheimers Dis. 2024;97(3):1289‐1298. doi:10.3233/JAD-230811 38217598

[19] Hinman JD , Elahi F , Chong D , et al. Placental growth factor as a sensitive biomarker for vascular cognitive impairment. Alzheimer's Dement. 2023;19(8):3519‐3527. doi:10.1002/alz.12974 36815663 PMC10440207

[20] Chong JR , Hilal S , Tan BY , et al. Clinical utility of plasma p‐tau217 in identifying abnormal brain amyloid burden in an Asian cohort with high prevalence of concomitant cerebrovascular disease. Alzheimer's Dement. 2025;21(2):e14502. doi:10.1002/alz.14502 39807654 PMC11848187

[21] Chong JR , Hilal S , Ashton NJ et al. Brain atrophy and white matter hyperintensities are independently associated with plasma neurofilament light chain in an Asian cohort of cognitively impaired patients with concomitant cerebral small vessel disease. Alzheimer's Dement (Amst). 2023;15(1):e12396. doi:10.1002/dad2.12396 36994314 PMC10040495

[22] Garcia‐Alloza M , Gregory J , Kuchibhotla KV , et al. Cerebrovascular lesions induce transient β‐amyloid deposition. Brain. 2011;134(12):3697‐3707. doi:10.1093/brain/awr300 22120142 PMC3235567

[23] Gaberel T , Gakuba C , Goulay R , et al. Impaired glymphatic perfusion after strokes revealed by contrast‐enhanced MRI. Stroke. 2014;45(10):3092‐3096. doi:10.1161/STROKEAHA.114.006617 25190438

[24] Martí‐Fàbregas J , Delgado‐Mederos R , Marín R , et al. Prognostic value of plasma β‐Amyloid levels in patients with acute intracerebral hemorrhage. Stroke. 2014;45(2):413‐417. doi:10.1161/STROKEAHA.113.002838 24385273

[25] Varela R , Gonzalez‐Ortiz F , Dias A , et al. Plasma brain‐derived tau in prognosis of large vessel occlusion ischemic stroke. Stroke. 2024;55(9):2353‐2358. doi:10.1161/STROKEAHA.123.046117 39051090

[26] Holmegaard L , Jensen C , Pedersen A , et al. Circulating levels of neurofilament light chain as a biomarker of infarct and white matter hyperintensity volumes after ischemic stroke. Sci Rep. 2024;14(1):16180. doi:10.1038/s41598-024-67232-1 39003344 PMC11246414

[27] Tony AA , Kholef EFM , Elgendy DB , Shoyb A . Neurofilament light chain correlates with stroke severity and clinical outcome in acute cerebrovascular stroke patients. Cell Mol Neurobiol. 2025;45(1):39. doi:10.1007/s10571-025-01552-2 40304765 PMC12044141

[28] Uphaus T , Bittner S , Gröschel S , et al. NfL (Neurofilament light chain) levels as a predictive marker for long‐term outcome after ischemic stroke. Stroke. 2019;50(11):3077‐3084. doi:10.1161/STROKEAHA.119.026410 31537188

[29] Pedersen A , Stanne TM , Nilsson S , et al. Circulating neurofilament light in ischemic stroke: temporal profile and outcome prediction. J Neurol. 2019;266(11):2796‐2806. doi:10.1007/s00415-019-09477-9 31375988 PMC6803587

[30] Pujol‐Calderón F , Portelius E , Zetterberg H , Blennow K , Rosengren LE , Höglund K . Neurofilament changes in serum and cerebrospinal fluid after acute ischemic stroke. Neurosci Lett. 2019;698:58‐63. doi:10.1016/j.neulet.2018.12.042 30599262

[31] Paul JF , Ducroux C , Correia P , et al. Serum glial fibrillary acidic protein in acute stroke: feasibility to determine stroke‐type, timeline and tissue‐impact. Front Neurol. 2024;15:1470718. doi:10.3389/fneur.2024.1470718 39777311 PMC11704488

[32] Barba L , Vollmuth C , Abu‐Rumeileh S , et al. Serum β‐synuclein, neurofilament light chain and glial fibrillary acidic protein as prognostic biomarkers in moderate‐to‐severe acute ischemic stroke. Sci Rep. 2023;13(1):20941. doi:10.1038/s41598-023-47765-7 38017278 PMC10684607

[33] Kumar A , Misra S , Yadav AK , et al. Role of glial fibrillary acidic protein as a biomarker in differentiating intracerebral haemorrhage from ischaemic stroke and stroke mimics: a meta‐analysis. Biomarkers. 2020;25(1):1‐8. doi:10.1080/1354750X.2019.1691657 31702405

[34] Chen Y , Nilsson AH , Goncalves I , et al. Evidence for a protective role of placental growth factor in cardiovascular disease. Sci Transl Med. 2020;12(572):eabc8587. doi:10.1126/scitranslmed.abc8587 33268513

[35] Che P , Wang S , Liu X , et al. Plasma placental growth factor as a biomarker for subcortical ischemic vascular dementia and its cognitive correlation mediated by white matter hyperintensities. Alzheimer's Dement. 2025;21(7):e70461. doi:10.1002/alz.70461 40622016 PMC12441591

[36] Wardlaw JM , Doubal F , Brown R , et al. Rates, risks and routes to reduce vascular dementia (R4vad), a UK‐wide multicentre prospective observational cohort study of cognition after stroke: protocol. Eur Stroke J. 2021;6(1):89‐101. doi:10.1177/2396987320953312 33817339 PMC7995325

[37] Wilcock D , Jicha G , Blacker D , et al. MarkVCID cerebral small vessel consortium: i. Enrollment, clinical, fluid protocols. Alzheimer's Dement. 2021;17(4):704‐715. doi:10.1002/alz.12215 33480172 PMC8122220

[38] Gruia D‐C , Trender W , Hellyer P , et al. IC3 protocol: a longitudinal observational study of cognition after stroke using novel digital health technology. BMJ Open. 2023;13(11):e076653. doi:10.1136/bmjopen-2023-076653 PMC10679983 38000822

[39] Page MJ , McKenzie JE , Bossuyt PM , et al. The PRISMA 2020 statement: an updated guideline for reporting systematic reviews. BMJ. 2021;372:n71. doi:10.1136/bmj.n71 33782057 PMC8005924

[40] Bernhardt J , Hayward KS , Kwakkel G , et al. Agreed definitions and a shared vision for new standards in stroke recovery research: the stroke recovery and rehabilitation roundtable taskforce. Int J Stroke. 2017;12(5):444‐450. doi:10.1177/1747493017711816 28697708

[41] Higgins JPT , Morgan RL , Rooney AA , et al. A tool to assess risk of bias in non‐randomized follow‐up studies of exposure effects (ROBINS‐E). Environ Int. 2024;186:108602. doi:10.1016/j.envint.2024.108602 38555664 PMC11098530

[42] McGuinness LA , Higgins JPT . Risk‐of‐bias VISualization (robvis): an R package and Shiny web app for visualizing risk‐of‐bias assessments. Res Synth Methods. 2021;12(1):55‐61. doi:10.1002/jrsm.1411 32336025

[43] Sterne JAC , Sutton AJ , Ioannidis JPA , et al. Recommendations for examining and interpreting funnel plot asymmetry in meta‐analyses of randomised controlled trials. BMJ. 2011;343:d4002. doi:10.1136/bmj.d4002 21784880

[44] Ferrari F , Rossi D , Ricciardi A , et al. Quantification and prospective evaluation of serum NfL and GFAP as blood‐derived biomarkers of outcome in acute ischemic stroke patients. J Cereb Blood Flow Metab. 2023;43(9):1601‐1611. doi:10.1177/0271678x231172520 37113060 PMC10414005

[45] Peng Y , Li Q , Qin L , et al. Combination of serum neurofilament light chain levels and MRI markers to predict cognitive function in ischemic stroke. Neurorehabil Neural Repair. 2021;35(3):247‐255. doi:10.1177/1545968321989354 33522401

[46] Gendron TF , Badi MK , Heckman MG , et al. Plasma neurofilament light predicts mortality in patients with stroke. Sci Transl Med. 2020;12(569):eaay1913. doi:10.1126/scitranslmed.aay1913 33177179 PMC9534269

[47] Egle M , Loubiere L , Maceski A , Kuhle J , Peters N , Markus HS . Neurofilament light chain predicts future dementia risk in cerebral small vessel disease. J Neurol Neurosurg Psychiatry. 2021;92(6):582‐589. doi:10.1136/jnnp-2020-325681 33558370 PMC8142459

[48] Sanchez E , Wilkinson T , Coughlan G , et al. Association of plasma biomarkers with cognition, cognitive decline, and daily function across and within neurodegenerative diseases: results from the ontario neurodegenerative disease research initiative. Alzheimer's Dement. 2024;20(3):1753‐1770. doi:10.1002/alz.13560 38105605 PMC10984487

[49] Stokowska A , Bunketorp Käll L , Blomstrand C et al. Plasma neurofilament light chain levels predict improvement in late phase after stroke. Eur J Neurol. 2021;28(7):2218‐2228. doi:10.1111/ene.14854 33811783

[50] Jiang L , Wang Z , Wang R , Li M , Zhang Y , Yang D . Plasma neurofilament light chain is associated with cognitive impairment after posterior circulation stroke. Evid Based Complement Alternat Med. 2022;2022:2466982. doi:10.1155/2022/2466982 35800005 PMC9256396

[51] Wang Z , Wang R , Li Y , et al. Plasma Neurofilament Light Chain as a Predictive Biomarker for Post‐stroke Cognitive Impairment: a Prospective Cohort Study. Front Aging Neurosci. 2021;13:631738. doi:10.3389/fnagi.2021.631738 33679379 PMC7933545

[52] Zheng P , Wang X , Chen J , Wang X , Shi SX , Shi K . Plasma Neurofilament Light Chain Predicts Mortality and Long‐Term Neurological Outcomes in Patients with Intracerebral Hemorrhage. Aging Dis. 2023;14(2):560‐571. doi:10.14336/AD.2022.21020 37008068 PMC10017162

[53] Wang J‐H , Huang J , Guo Fu‐Q , et al. Circulating neurofilament light predicts cognitive decline in patients with post‐stroke subjective cognitive impairment. Front Aging Neurosci. 2021;13:665981. doi:10.3389/fnagi.2021.665981 34079450 PMC8165181

[54] Li H , Yang D , Liu S , et al. Effects of early antihypertensive treatment on cognitive function in patients with acute ischemic stroke with different neurofilament light chain levels. J Stroke Cerebrovasc Dis. 2025;34(2):108206. doi:10.1016/j.jstrokecerebrovasdis.2024.108206 39708937

[55] Chen HG , Wang M , Jiao AH , et al. Research on changes in cognitive function, β‐amyloid peptide and neurotrophic factor in stroke patients. Eur Rev Med Pharmacol Sci. 2018;22(19):6448‐6455. doi:10.26355/eurrev_201810_16057 30338813

[56] Shi X , Zhang X , Ao JF , Yang M . Correlation between Non‐HDL‐C/HDL‐C and Aβ1‐42 levels in cerebral infarction‐related cognitive dysfunction. Clin Neurol Neurosurg. 2024;245:108503. doi:10.1016/j.clineuro.2024.108503 39178633

[57] Huang K‐L , Hsiao I‐T , Chang T‐Yu , et al. Neurodegeneration and vascular burden on cognition after midlife: a plasma and neuroimaging biomarker study. Front Hum Neurosci. 2021;15:735063. doi:10.3389/fnhum.2021.735063 34970128 PMC8712753

[58] Chi N‐F , Chao S‐P , Huang Li‐K , et al. Plasma amyloid beta and tau levels are predictors of post‐stroke cognitive impairment: a longitudinal study. Front Neurol. 2019;10:715. doi:10.3389/fneur.2019.00715 31312178 PMC6614443

[59] Tang S‐C , Yang K‐C , Chen C‐H , et al. Plasma β‐Amyloids and tau proteins in patients with vascular cognitive impairment. Neuromolecular Med. 2018;20(4):498‐503. doi:10.1007/s12017-018-8513-y 30242618

[60] Huang Li‐K , Chao S‐P , Hu C‐J , Chien Li‐N , Chiou H‐Yi , Lo Yu‐C , Hsieh Yi‐C Plasma Phosphorylated‐tau181 is a predictor of post‐stroke cognitive impairment: a longitudinal study. Front Aging Neurosci. 2022;14:889101. doi:10.3389/fnagi.2022.889101 35572134 PMC9099290

[61] Mao L , Chen X‐H , Zhuang J‐H , et al. Relationship between β‐amyloid protein 1‐42, thyroid hormone levels and the risk of cognitive impairment after ischemic stroke. World J Clin Cases. 2020;8(1):76‐87. doi:10.12998/wjcc.v8.i1.76 31970172 PMC6962069

[62] Giudici KV , de Souto Barreto P , Guyonnet S , Li Y , Bateman RJ , Vellas B Assessment of plasma amyloid‐β42/40 and cognitive decline among community‐dwelling older adults. JAMA Netw Open. 2020;3(12):e2028634. doi:10.1001/jamanetworkopen.2020.28634 33331917 PMC7747018

[63] Trelle AN , Young CB , Vossler H , et al. Plasma Aβ42/Aβ40 is sensitive to early cerebral amyloid accumulation and predicts risk of cognitive decline across the Alzheimer's disease spectrum. Alzheimer's Dement. 2025;21(2):e14442. doi:10.1002/alz.14442 39713875 PMC11848181

[64] Mielke MM , Hagen CE , Wennberg AMV , et al. Association of plasma total tau level with cognitive decline and risk of mild cognitive impairment or dementia in the mayo clinic study on aging. JAMA Neurol. 2017;74(9):1073‐1080. doi:10.1001/jamaneurol.2017.1359 28692710 PMC5710182

[65] Sturchio A , Dwivedi AK , Malm T , et al. High Soluble Amyloid‐β42 Predicts Normal Cognition in Amyloid‐Positive Individuals with Alzheimer's Disease‐Causing Mutations. J Alzheimers Dis. 2022;90(1):333‐348. doi:10.3233/JAD-220808 36120786 PMC9661329

[66] Zhang Y , Bander ED , Lee Y , Muoser C , Schaffer CB , Nishimura N . Microvessel occlusions alter amyloid‐beta plaque morphology in a mouse model of Alzheimer's disease. J Cereb Blood Flow Metab. 2020;40(10):2115‐2131. doi:10.1177/0271678x19889092 31744388 PMC7786844

[67] Moulin S , Leys D , Schraen‐Maschke S et al. Aβ1‐40 and Aβ1‐42 plasmatic levels in stroke: influence of pre‐existing cognitive status and stroke characteristics. Curr Alzheimer Res. 2017;14(6):686‐694. doi:10.2174/1567205012666151027141730 26502812

[68] Barthélemy NR , Horie K , Sato C , Bateman RJ . Blood plasma phosphorylated‐tau isoforms track CNS change in Alzheimer's disease. J Exp Med. 2020;217(11):e20200861. doi:10.1084/jem.20200861 32725127 PMC7596823

[69] Gonzalez‐Ortiz F , Turton M , Kac PłR , et al. Brain‐derived tau: a novel blood‐based biomarker for Alzheimer's disease‐type neurodegeneration. Brain. 2023;146(3):1152‐1165. doi:10.1093/brain/awac407 36572122 PMC9976981

[70] Gundersen JK , Gonzalez‐Ortiz F , Karikari T , et al. Neuronal plasma biomarkers in acute ischemic stroke. J Cereb Blood Flow Metab. 2025;45(1):77‐84. doi:10.1177/0271678x241293537 39450480 PMC11563507

[71] Shan W , Jiang R , Wang J , Xu G , Zhao J , Zhai G , Shao J High serum glial fibrillary acidic protein levels are associated with increased risk of post‐stroke cognitive impairment. Front Aging Neurosci. 2025;17:1546270. doi:10.3389/fnagi.2025.1546270 40421099 PMC12104187

[72] Kern KC , Vohra M , Thirion ML , et al. White matter free water mediates the associations between placental growth factor, white matter hyperintensities, and cognitive status. Alzheimer's Dement. 2025;21(2):e14408. doi:10.1002/alz.14408 39692213 PMC11848340

[73] Jaime Garcia D , Clancy U , Arteaga C , et al. Blood biomarkers of vascular dysfunction in small vessel disease progression: insights from a longitudinal neuroimaging study. Alzheimer's Dement. 2025;21(4):e70152. doi:10.1002/alz.70152 40275856 PMC12022501

[74] Sawano A , Takahashi T , Yamaguchi S , Aonuma M , Shibuya M . Flt‐1 but not KDR/Flk‐1 tyrosine kinase is a receptor for placenta growth factor, which is related to vascular endothelial growth factor. Cell Growth Differ. 1996;7(2):213‐221. 8822205

[75] Ziche M , Maglione D , Ribatti D , et al. Placenta growth factor‐1 is chemotactic, mitogenic, and angiogenic. Lab Invest. 1997;76(4):517‐531. 9111514

[76] Odorisio T , Schietroma C , Zaccaria ML , et al. Mice overexpressing placenta growth factor exhibit increased vascularization and vessel permeability. J Cell Sci. 2002;115(12):2559‐2567. doi:10.1242/jcs.115.12.2559 12045226

[77] Escudero C , Acurio J , López E , et al. Vascular endothelial growth factor and poor prognosis after ischaemic stroke. Eur J Neurol. 2021;28(5):1759‐1764. doi:10.1111/ene.14641 33176035

[78] Ball EL , Sutherland R , Squires C , et al. Predicting post‐stroke cognitive impairment using acute CT neuroimaging: a systematic review and meta‐analysis. Int J Stroke. 2022;17(6):618‐627. doi:10.1177/17474930211045836 34569865 PMC9260488

[79] Ball EL , Shah M , Ross E et al. Predictors of post‐stroke cognitive impairment using acute structural MRI neuroimaging: a systematic review and meta‐analysis. Int J Stroke. 2023;18(5):543‐554. doi:10.1177/17474930221120349 35924821 PMC10201083

[80] Omran MSL , Ibrahim NHM , Zaki MA . Cognitive impairment after first‐ever ischemic stroke. Al‐Azhar Assiut Med J. 2022;20(4):338. doi:10.4103/azmj.azmj_72_21

[81] Panikkar D , Vivek S , Crimmins E , Faul J , Langa KM , Thyagarajan B . Pre‐analytical variables influencing stability of blood‐based biomarkers of neuropathology. J Alzheimers Dis. 2023;95(2):735‐748. doi:10.3233/JAD-230384 37574735 PMC11520930

[82] Li CY , Fan LY , Lin CH , Hu CJ , Chiu MJ . Ultrasensitive assays detect different conformations of plasma β amyloids. ACS Omega. 2025;10(7):7256‐7263. doi:10.1021/acsomega.4c10879 40028141 PMC11865983

[83] Nasreddine ZS , Phillips NA , Bédirian V , et al. The montreal cognitive assessment, MoCA: a brief screening tool for mild cognitive impairment. J Am Geriatr Soc. 2005;53(4):695‐699. doi:10.1111/j.1532-5415.2005.53221.x 15817019

[84] Folstein MF , Folstein SE , McHugh PR . “Mini‐mental state”: a practical method for grading the cognitive state of patients for the clinician. J Psychiatr Res. 1975;12(3):189‐198. doi:10.1016/0022-3956(75)90026-6 1202204

[85] Coen RF , Robertson DA , Kenny RA , King‐Kallimanis BL . Strengths and limitations of the MoCA for assessing cognitive functioning: findings from a large representative sample of irish older adults. J Geriatr Psychiatry Neurol. 2016;29(1):18‐24. doi:10.1177/0891988715598236 26251108

[86] Gruia DC , Giunchiglia V , Coghlan A , et al. Development and validation of the IC3: an online remote assessment technology for deep phenotyping and monitoring of cognitive impairment after stroke. Assessment. Published online October 28, 2025. doi:10.1177/10731911251381572 PMC13675564 41147646

[87] Kim KY , Shin KY , Chang KA . Potential biomarkers for post‐stroke cognitive impairment: a systematic review and meta‐analysis. Int J Mol Sci. 2022;23(2):602. doi:10.3390/ijms23020602 35054785 PMC8775398

[88] Ma Y , Chen Y , Yang T , et al. Blood biomarkers for post‐stroke cognitive impairment: a systematic review and meta‐analysis. J Stroke Cerebrovasc Dis. 2024;33(8):107632. doi:10.1016/j.jstrokecerebrovasdis.2024.107632 38417566

[89] Duering M , Biessels GJ , Brodtmann A , et al. Neuroimaging standards for research into small vessel disease—advances since 2013. Lancet Neurol. 2023;22(7):602‐618. doi:10.1016/S1474-4422(23)00131-X 37236211

[90] Kerkhofs D , Wong SM , Zhang E , et al. Blood–brain barrier leakage at baseline and cognitive decline in cerebral small vessel disease: a 2‐year follow‐up study. GeroScience. 2021;43(4):1643‐1652. doi:10.1007/s11357-021-00399-x 34160780 PMC8492799

[91] Braban A , O'Hanlon H , Gruia D‐C et al. What can we learn from microstructural properties of stroke lesions in relation to cognitive outcomes after stroke? (S2.009). Neurology. 2025;104(7_Supplement_1):2213. doi:10.1212/WNL.0000000000208993

[92] Zhang B , Zhang C , Wang Y , et al. Effect of renal function on the diagnostic performance of plasma biomarkers for Alzheimer's disease. Front Aging Neurosci. 2023;15:1150510. doi:10.3389/fnagi.2023.1150510 37009461 PMC10050758

[93] Berry K , Asken BM , Grab JD , et al. Hepatic and renal function impact concentrations of plasma biomarkers of neuropathology. Alzheimer's Dement (Amst). 2022;14(1):e12321. doi:10.1002/dad2.12321 35845260 PMC9274803

[94] Huber H , Ashton NJ , Schieren A , et al. Levels of Alzheimer's disease blood biomarkers are altered after food intake—a pilot intervention study in healthy adults. Alzheimer's Dement. 2023;19(12):5531‐5540. doi:10.1002/alz.13163 37243891

