# Supplementary material for: Dementia blood biomarkers in the context of post‐stroke cognitive outcomes: Systematic review and evidence synthesis
Source: Alzheimers Dement. 2026 Jul 6;22(7):e71653. doi: 10.1002/alz.71653 (PMC13337546; doi:10.1002/alz.71653)
Supplement: Supplementary file 2 — Supporting Information [file ALZ-22-e71653-s002.docx]

# Supplementary Material 1: Search Strategy

In collaboration with a research librarian, search strategies for the systematic review were developed. Databases searched included MEDLINE, Embase, Web of Science, and Scopus. The searches were all run on 13^th^ March 2025 with no restrictions on publication date.

Orange = stroke populations

Purple = cognition as outcome

Green = Protein name

Blue = Blood-derived biomarkers

Red = exclusion terms

## OVID Medline:

| Line | Search Statement |
| --- | --- |
| 1 | exp Stroke/ 189373 |
| 2 | (Stroke* or Cerebrovascular accident* or Cerebrovascular disease*).mp. [mp=title, book title, abstract, original title, name of substance word, subject heading word, floating sub-heading word, keyword heading word, organism supplementary concept word, protocol supplementary concept word, rare disease supplementary concept word, unique identifier, synonyms, population supplementary concept word, anatomy supplementary concept word] 447266 |
| 3 | 1 or 2 469959 |
| 4 | exp Dementia, Vascular/ 7928 |
| 5 | (cognition or cognitive or Cognitive impairment* or cognitively impaired or cognitive decline or VCI or post stroke cognitive impairment or Post-stroke cognitive impairment or PSCI or PSCID or Vascular dementia* or Mixed dementia*).mp. [mp=title, book title, abstract, original title, name of substance word, subject heading word, floating sub-heading word, keyword heading word, organism supplementary concept word, protocol supplementary concept word, rare disease supplementary concept word, unique identifier, synonyms, population supplementary concept word, anatomy supplementary concept word] 654002 |
| 6 | 4 or 5 656442 |
| 7 | Neurofilament Proteins/ 6705 |
| 8 | Amyloid beta-Peptides/ 44435 |
| 9 | tau Proteins/ 21410 |
| 10 | Placenta Growth Factor/ 2640 |
| 11 | Glial Fibrillary Acidic Protein/ 17413 |
| 12 | (neurofilament light or neurofilament protein or NfL or glial fibrillary acidic protein or GFAP or Phosphorylated tau or tau protein or ptau or ptau181 or p-tau181 or ptau-181 or ptau217 or p-tau217 or ptau-217 or Amyloid beta or Placenta* growth factor or PlGF or PGF).mp. [mp=title, book title, abstract, original title, name of substance word, subject heading word, floating sub-heading word, keyword heading word, organism supplementary concept word, protocol supplementary concept word, rare disease supplementary concept word, unique identifier, synonyms, population supplementary concept word, anatomy supplementary concept word] 123703 |
| 13 | 7 or 8 or 9 or 10 or 11 or 12 133433 |
| 14 | 3 and 6 and 13 750 |
| 15 | exp "review"/ 3453648 |
| 16 | 14 not 15 581 |
| 17 | Biomarkers/ 380062 |
| 18 | (Blood or serum or plasma).mp. [mp=title, book title, abstract, original title, name of substance word, subject heading word, floating sub-heading word, keyword heading word, organism supplementary concept word, protocol supplementary concept word, rare disease supplementary concept word, unique identifier, synonyms, population supplementary concept word, anatomy supplementary concept word] 5380120 |
| 19 | 17 and 18 232580 |
| 20 | 16 and 19 68 |

## Embase:

| Line | Search Statement |
| --- | --- |
| 1 | exp Stroke/ 488848 |
| 2 | (Stroke* or Cerebrovascular accident* or Cerebrovascular disease*).mp. [mp=title, abstract, heading word, drug trade name, original title, device manufacturer, drug manufacturer, device trade name, keyword heading word, floating subheading word, candidate term word] 802699 |
| 3 | 1 or 2 802699 |
| 4 | exp Dementia, Vascular/ 15901 |
| 5 | (cognition or cognitive or Cognitive impairment* or cognitively impaired or cognitive decline or VCI or post stroke cognitive impairment or Post-stroke cognitive impairment or PSCI or PSCID or Vascular dementia* or Mixed dementia*).mp. [mp=title, abstract, heading word, drug trade name, original title, device manufacturer, drug manufacturer, device trade name, keyword heading word, floating subheading word, candidate term word] 989940 |
| 6 | 4 or 5 992796 |
| 7 | Neurofilament Proteins/ 8211 |
| 8 | Amyloid beta-Peptides/ 59315 |
| 9 | tau Proteins/ 43681 |
| 10 | Placenta Growth Factor/ 7907 |
| 11 | Glial Fibrillary Acidic Protein/ 40148 |
| 12 | (neurofilament light or neurofilament protein or NfL or glial fibrillary acidic protein or GFAP or Phosphorylated tau or tau protein or ptau or ptau181 or p-tau181 or ptau-181 or ptau217 or p-tau217 or ptau-217 or Amyloid beta or Placenta* growth factor or PlGF or PGF).mp. [mp=title, abstract, heading word, drug trade name, original title, device manufacturer, drug manufacturer, device trade name, keyword heading word, floating subheading word, candidate term word] 200242 |
| 13 | 7 or 8 or 9 or 10 or 11 or 12 200242 |
| 14 | 3 and 6 and 13 2558 |
| 15 | exp "review"/ 3461090 |
| 16 | 14 not 15 1815 |
| 17 | Biomarkers/ 526797 |
| 18 | (Blood or serum or plasma).mp. [mp=title, abstract, heading word, drug trade name, original title, device manufacturer, drug manufacturer, device trade name, keyword heading word, floating subheading word, candidate term word] 8086858 |
| 19 | 17 and 18 241982 |
| 20 | 16 and 19 208 |

## Web of science:

Core collection included:

*- WOS.SCI (Science citation index): 1970 to 2025*

*- WOS.AHCI (Arts and humanities citation index): 1975 to 2025*

*- WOS.ESCI (Emerging sources citation index): 2015 to 2025*

*- WOS.ISTP (Index to scientific & technical proceedings): 1990 to 2025*

*- WOS.SSCI(Social sciences citation index): 1970 to 2025*

*- WOS.ISSHP: 1990 to 2025*

| Line | Search Statement |
| --- | --- |
| 1 | *(TS=(biomarker*)) OR (TI=(blood)) OR (TI=(serum)) OR (TI=(plasma))OR (AB=(biomarker*)) OR (AB=(blood)) OR (AB=(serum)) OR (AB=(plasma)) Results: 4951554* |
| 2 | *TS=(stroke*)) OR (TI=(cerebrovascular accident*)) OR (TI=(cerebrovascular disease*))OR (AB=(stroke*)) OR (AB=(cerebrovascular accident*)) OR (AB=(cerebrovascular disease*)) Results: 534148* |
| 3 | *(TS=(Cogniti*)) OR (TS=(VCI)) OR (TS=(PSCID)) OR (TS=("Vascular dementia")) OR (TS=("Mixed dementia")) OR (TS=("PSCI")) OR (TS=("post stroke cognitive impairment")) OR (TS=("post-stroke cognitive impairment"))OR (AB=(Cogniti*)) OR (AB=(VCI)) OR (AB=(PSCID)) OR (AB=("Vascular dementia")) OR (AB=("Mixed dementia")) OR (AB=("PSCI")) OR (AB=("post stroke cognitive impairment")) OR (AB=("post-stroke cognitive impairment")) Results: 1065719* |
| 4 | *(TI=("Placenta growth factor")) OR (TI=("PlGF")) OR (TI=("PGF")) OR (AB=("Placenta growth factor")) OR (AB=("PlGF")) OR (AB=("PGF")) Results: 13706* |
| 5 | *TI=("Amyloid beta")) OR (TI=(Amyloid)) OR (AB=("Amyloid beta")) OR (AB=(Amyloid)) Results: 119913* |
| 6 | *(TI=("phosphorylated tau")) OR (TI=(tau protein)) OR (TI=("ptau")) OR (TI=("ptau-181")) OR (TI=("ptau181")) OR (TI=("p-tau181")) OR (TI=("ptau-217")) OR (TI=("ptau217")) OR (TI=("p-tau217")) OR (AB=("phosphorylated tau")) OR (AB=(tau protein)) OR (AB=("ptau")) OR (AB=("ptau-181")) OR (AB=("ptau181")) OR (AB=("p-tau181")) OR (AB=("ptau-217")) OR (AB=("ptau217")) OR (AB=("p-tau217")) Results: 29264* |
| 7 | *(TI=("Glial Fibrillary Acidic Protein")) OR (TI=(GFAP)) OR (AB=("Glial Fibrillary Acidic Protein")) OR (AB=(GFAP)) Results: 28216* |
| 8 | *(TI=("neurofilament light")) OR (TI=(NfL)) OR (TI=("Neurofilament protein")) OR (AB=("neurofilament light")) OR (AB=(NfL)) OR (AB=("Neurofilament protein")) Results: 9820* |
| 9 | *#1 AND #2 AND #3 Results: 6638* |
| 10 | *: #8 OR #7 OR #6 OR #5 OR #4 Results: 185448* |
| 11 | *#9 AND #10 Results: 865* |

## Scopus:

| Line | Search Statement |
| --- | --- |
| 1 | TITLE-ABS-KEY(Stroke* OR "cerebrovascular accident*" OR "cerebrovascular disease*") |
|  | ***AND*** |
| 2 | *TITLE-ABS-KEY(cognition OR cognitive OR "cognitive impairment*" OR "cognitively impaired" OR "cognitive decline" OR VCI OR "post stroke cognitive impairment" OR "post-stroke cognitive impairment" OR PSCI OR PSCID OR "vascular dementia*" OR "mixed dementia*")* |
|  | ***AND*** |
| 3 | TITLE-ABS-KEY("neurofilament light" OR "neurofilament protein" OR NfL OR "glial fibrillary acidic protein" OR GFAP OR "phosphorylated tau" OR "tau protein" OR ptau OR ptau181 OR "p-tau181" OR ptau-181 OR ptau217 OR "p-tau217" OR ptau-217 OR "Amyloid beta" OR "Placenta* growth factor" OR PlGF OR PGF) |
|  | **AND** |
| 4 | *TITLE-ABS-KEY(blood OR serum OR plasma)* |
|  | ***AND*** |
| 5 | *EXCLUDE(DOCTYPE, "re") AND EXCLUDE(DOCTYPE, "er") AND EXCLUDE(DOCTYPE, "sh") AND EXCLUDE(DOCTYPE, "tb") AND EXCLUDE(DOCTYPE, "no") AND EXCLUDE(DOCTYPE, "ch") AND EXCLUDE(DOCTYPE, "le") AND EXCLUDE(DOCTYPE, "cp")* |
