# Supplementary material for: Dementia blood biomarkers in the context of post‐stroke cognitive outcomes: Systematic review and evidence synthesis
Source: Alzheimers Dement. 2026 Jul 6;22(7):e71653. doi: 10.1002/alz.71653 (PMC13337546; doi:10.1002/alz.71653)
Supplement: Supplementary file 3 — Supporting Information [file ALZ-22-e71653-s004.docx]

# Supplementary Material 2: Data extraction template

**General information**

Title

Title of paper / abstract / report that data are extracted from

Lead author

Can either put the in text citation or full reference

Journal

Impact Factor

Notes

Any information from the introduction that provides important context.

Aim(s) of study

Usually found just before the start of the methods.

# Methods

Study design

Longitudinal studies are typically cohort studies.

1.
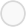
 Cohort study
2.
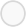
 Cross sectional study
3.
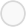
 Case control study
4.
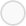
 Case series
5.
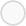
 Diagnostic test accuracy study
6.
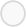
 Clinical prediction rule
7.
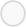
 Other Location of study site
8.
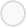
 United States
9.
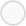
 UK
10.
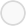
 China
11.
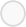
 Canada
12.
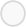
 Australia
13.
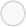
 Other

# Participant information

Inclusion criteria

Exclusion criteria

Time Frame of Study

**Start date End date**

**Duration of study Follow-up period(s)**

# Data metrics

**Date/Time**

When was data collected?

Mark an X if data was collected at that time point. typically 0 month is stroke incidence.

**Blood Biomarker(s) Cognitive test(s) Imaging Functional scores (mRS. NIHSS etc)**

**0 month**

**3 month**

**6 month**

**9 month**

**1 year**

**> 1 year Other**

Notes on data collection Explicit information if required.

# Biomarker data details

Plasma Biomarker measured

1.
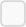
 Neurofilament light (NfL)
2.
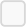
 Glial Fibrillary Acidic Protein
3.
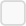
 Amyloid beta (40 or 42)
4.
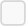
 Tau (total or phosphorylated)
5.
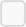
 Placental Growth Factor 6.


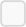


Other

Specific biomarker name(s)

e.g. Ab 42

# Measurement methodology and units

Fasted blood?

1.
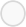
 Yes
2.
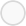
 No
3.
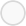
 Unknown Notes

Pre-processing of bloods

Information on pre-processing of bloods (if data available).e.g. centrifuged at X × g at Y°C for Z minutes.

If unknown: N/A

Blood analysis method

Analysis method: typically ELISA's, Immunomagnetic reduction (IMR), or SIngle MOlecular Array (SIMOA).

If unknown: N/A

Cut-off values/thresholds If unknown: N/A

# Cognitive outcomes

Type of Cognitive test

- Look to see if it was Adjusted/Corrected based on other factors (e.g. education level) .

e.g MoCA (adjusted for X,Y,Z) or MoCA (non-corrected)

How did they define cognitively impaired?

e.g. Moca <26 = cognitively impaired

# Stroke Characteristics

Stroke etiology

Small vessel disease, large vessel disease etc.

Stroke location(s)

Potentially put the percentage of each type? If unknown: N/A

Other co-variates collected

1.
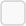
 Education
2.
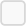
 Fazekas score
3.
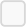
 NIHSS
4.
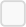
 mRS
5.
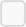
 Hypertension
6.
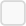
 Diabetes Mellitus
7.
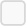
 Smoking
8.
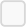
 Drinking
9.
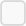
 Hyperlipidemia
10.
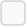
 Renal Markers (e.g. eGFR) Other

# Statistical tests

Mean values // Correlations // Regression analysis // ROC/AUC analysis

# Results

Population Characteristics Report the numbers!

Make a note in the comment box for which groups are being compared. e.g. CG1 = non-PSCI. CG2 = PSCI

If unknown (but should be present): Unknown If not applicable: N/A

**Total sample size**

**Gender distribution (M/F) Median/mean age Education**

**Fazeka's Scoring (mean +/-or median IQR)**

**Volume of stroke mRS NIHSS**

**NfL GFAP**

**total tau p-tau181**

**Comparison group 1**

**Comparison group 2**

**Comparison group 3**

**Comparison group 4**

**p-tau217 Aβ40 Aβ42**

**Aβ40/42 ratio**

Extra notes

**Comparison group 1**

**Comparison group 2**

**Comparison group 3**

**Comparison group 4**

(E.g. how did they measure education?)

# Primary Findings

Cross-sectional Associations between biomarker levels and cognitive performance

association of biomarkers with cognitive scores when measured at the same (or near enough) timepoint. Include:

- means, medians, for biomarker and cognitive scores
- correlation coefficients
- simple or multivariable linear'/logistic regression
- effect sizes

Longitudinal prediction of cognitive outcomes from biomarker levels Includes:

- logistic or linear regression
- ROC curves with AUC values

Other analysis / results non involving biomarkers

Other information(s)
