# Supplementary material for: Dementia blood biomarkers in the context of post‐stroke cognitive outcomes: Systematic review and evidence synthesis
Source: Alzheimers Dement. 2026 Jul 6;22(7):e71653. doi: 10.1002/alz.71653 (PMC13337546; doi:10.1002/alz.71653)
Supplement: Supplementary file 4 — Supporting Information [file ALZ-22-e71653-s005.docx]

# Supplementary Material 3: Additional characteristics of screened studies

This table summarises study‑level characteristics including pre‑analytical procedures, biomarkers measured, inclusion and exclusion criteria, stroke aetiology, baseline medical history, clinical and imaging variables collected, and covariates included in adjusted statistical models. Information on the number of prior strokes was not systematically reported across included studies; where prior stroke was an exclusion criterion, this is noted in the exclusion criteria column.

| Study ID | Preanalytical procedures | Biomarker Measured | Inclusion criteria | Exclusion criteria | Stroke aetiology | Baseline medical history, clinical, and imaging variables collected | Covariates included in adjusted statistical models |
| --- | --- | --- | --- | --- | --- | --- | --- |
| Chen et al. (2018) [China] | 4000× g at 4°C for 10 min (ELISA).  12000× g at 4°C for 10 min (Western blot). | Aβ40 | (1) Patients diagnosed with first onset acute stroke by the director of department based on the guidelines for the diagnosis of stroke via magnetic resonance imaging (MRI) or computed tomography (CT) (2) Patients without sensory deficits (hearing or visual) to complete study tests  (3) Patients without severe motor dysfunction (4) Patients without serious systemic complications (malignant tumour, hepatic and renal dysfunction, blood system diseases, etc.) | (1) Subjects who quit midway  (2) Subjects receiving emergency thrombolysis or interventional therapy  (3) Patients who were unable to complete the experiment due to the deterioration of disease  (4) Subjects suffering from Alzheimer's Disease  PLEASE NOTE: only exclusion for Alzheimer's Disease and no mention of exclusion for other diagnosed neurodegenerative disease. | Not reported | Education;Body mass index (BMI) | No covariate adjustment reported |
| Chi et al, (2019) [Taiwan] | Centrifuged at 1500 ×g at unknown temperature for 15 minutes.  Stored at 80°C. | Aβ (40 and 42), total tau | (1) Adult patients ≥ 20 years old  (2) Admitted to hospital within 7 days of acute ischaemic stroke | (1) Patients with known cognitive impairment or a neurodegenerative disease that impaired regular daily activities before a stroke  (2) Patients with large infarcts (≥1/3 middle cerebral artery territory) that caused immediate consciousness or cognitive impairment after a stroke  (3) Patients with strategic infarcts involving the paramedian thalamus, hippocampus, or medial frontal cortex with a high risk of cognitive impairment  (4) Patients with severe language or physical disability that impeded neuropsychological testing  (5) The patients who could not complete the MoCA test at 3 months due to physical disability (e.g., dominant hand weakness and unable to complete the drawing tasks) were excluded at 3 months (n = 5) | Various aetiologies including:  large artery atherosclerosis  small-vessel disease  cardioembolism  undetermined aetiology  A large proportion of participants had small-vessel disease (41/55) | Education ; Fazekas score ; NIHSS; Hypertension ; Diabetes Mellitus ; Hyperlipidemia | No covariate adjustment reported |
| Egle et al. (2021) | Not reported | NfL | (1) Symptomatic SVD, defined as clinical lacunar stroke syndrome  (2) Confluent regions of WMH graded Fazekas score ≥2  (3) At least 3 months post-stroke | (1) Stroke not caused by SVD  (2) Other central nervous system diseases  (3) Major psychiatric disorders  (4) Other causes of White matter disease other than SVD | Small vessel disease | Education; Fazekas score; mRS; premorbid IQ (National Adult Reading Test) | Age; sex/gender; premorbid IQ (National Adult Reading Test); DTI‑derived MDPH |
| Ferrari et al. (2023) | Venous blood centrifuged at 2000 × g at 24°C for 10 minutes within two hours of collection. Stored in -80°C within cryovials. | GFAP, NfL | (1) Patients admitted to the Stroke Unit department of the Policlinico San Matteo, Pavia, between August 2019 and March 2021  (2) Patients of both sexes aged more than 18 years  (3) < 24 hours after ischemic stroke symptom onset, with scoring via the National Institute of Health Stroke Scale (NIHSS) > or equal to 1 | (1) Previous clinically symptomatic ischaemic or haemorrhagic stroke  (2) Previous traumatic head injuries with residual deficits  (3) active CNS or PNS disease other than CVD  (4) Cancer disease with life expectancy < 12 months  (5) Pregnancy  (6) Haemorrhagic stroke | Atherosclerosis of large caliber arteries: N=13 (36.1%)  Cardio-aortic embolism: N= 15 (41.7%)  Occlusion of small vessels: N= 3 (8.3%)  Indeterminate cause: N= 2 (5.6%)  Other cause: N= 1 (2.8%)  Not known: N= 2 (5.5%) | Education; Fazekas score; NIHSS; mRS; hypertension; smoking; hyperlipidaemia; pre‑existing comorbidities (presence/absence reported, not further specified) | Age; mRS; CCVB (CT‑adapted Fazekas scale); DTI‑derived MDPH; blood biomarkers (NFL, GFAP) |
| Gendron et al. (2020) | Plasma samples were centrifuged at 10,000 × g at unknown temperature for 5 min before transferring samples to 96-well plates along with calibrators and quality control samples. | NfL | (1) Participants in the Mayo Clinic Florida Familial Cerebrovascular Diseases Registry  (2) Aged 18 years and older along with consenting adult  (3) Participants were diagnosed as having definite acute ischemic stroke if they had symptoms of stroke and a corresponding lesion on brain imaging showing focal infarction  (4) All CVD-affected individuals were assigned a neurologist-confirmed primary diagnosis | Not specifically stated | SAH (subarachnoid haemorrhage: N=58  Non-traumatic ICH (intercerebral haemorrhage): N= 29  ACI (acute cerebral infarction): N=227  TOAST:  large-artery atherosclerosis (LAA): N=47  small-vessel occlusion (SVO): N=15  cardioembolism (CE): N=87  stroke of undetermined aetiology(UDE): N=70  stroke of other determined aetiology (ODE): N=8 | NIHSS; mRS; hypertension; diabetes mellitus; smoking; BMI; physical activity; race | Time from stroke to blood draw; age at blood draw; sex; BMI; current smoking; hypertension; diabetes mellitus; physical activity |
| Huang et al. (2021) | 3000 × g at unknown °temperature for 20 minutes. Stored at -80°C. | Aβ (40 and 42), total tau | (1) A diagnosis of acute ischemic stroke confirmed on magnetic resonance imaging (MRI) at stroke onset ~ 3 months after onset  (2) Education years at least 6 years  (3) No history of old stroke, dementia, tauopathy diseases, substantial traumatic brain injury or epilepsy before the index stroke  (4) Without recurrent stroke occurring between the index stroke and the study screening procedure  (5) Without persistent moderate to severe dysphasia, which was defined as a score of > 1 point in the language score of the National Institutes of Health Stroke Scale  In addition, age- and education-matched elderly normal controls were also recruited:  (1) Education at least 6 years  (2) No subjective cognitive complaint  (3) No major neurological and psychiatric disease  (4) The sum of Clinical Dementia Rating sub-scores was 0 | Not specifically stated | Ischemic stroke (unknown TOAST scores) | Education; NIHSS; hypertension; diabetes mellitus; gout; dyslipidaemia; APOE4 genotyping | Age; education; depressive symptoms (GDS) |
| Huang et al. (2022) | Centrifuged 1500×g for 15min at room temperature (transferred to EDTA tube [0.5ml]). Stored at -80°C until biomarker assays. | Aβ (40 and 42), Tau (total and p-tau181) | (1) Patients aged ≥ 20 years who were admitted to Shuang-Ho Hospital, Taipei Medical University  (2) Within 7 days of acute ischemic stroke | (1) Premorbid cognitive impairment, mood disorders, or neurodegenerative diseases that impaired daily activities  (2) Large infarcts causing immediate consciousness impairment  (3) Strategic infarcts involving the hippocampus or medial frontal cortex  (4) Severe language or physical disabilities that hinder neuropsychological testing | TOAST classification:  Large artery atherosclerosis, n = 23 (N=136)  Small vessel occlusion, n = 85 (N=136)  Cardioembolism, n = 13 (N=136)  Specific etiology, n = 4 (N=136)  Undetermined etiology, n = 9 (N=136) | Education; Fazekas score; NIHSS; hypertension; diabetes mellitus; smoking; drinking; hyperlipidaemia; BMI; microbleed anatomical rating scale | Education; hypertension |
| Jiang et al. (2022) | Centrifuged at 3000 × g at room temp °C for 20 minutes.  Samples were then stored at -80°C. | NfL | (1) 18+  (2) First ever Acute ischaemic stroke of the posterior circulation  (3) Within 24 hours of symptom onset  (4) Confirmed with CT or MRI according to WHO guidelines | (1) Dementia or considerable cognitive impairment before the stroke  (2) Mental disorders, or being unable to complete the cognitive exams  (3) Major neurological illness other than stroke (AD, PD)  (4) Autoimmune, haematologic illnesses, severe hepatic, renal, or thyroid problems, or cardiac failure | Characterised by the TOAST criteria in this order:  Total, PSCI, non-PSCI  Large-artery atherosclerosis  151 (57.20%) // 64 (63.37%) // 87 (53.37%)  Cardioembolism  44 (16.67%) // 16 (15.84%) // 28 (17.18%)  Small vessel occlusion  23 (8.71%) // 9 (8.91%) // 14 (8.59%)  Other cause  18 (6.82%) // 7 (6.93%) // 11 (6.75%)  Undetermined  28 (10.61%) // 5 (4.95%) // 23 (14.11%) | Education; NIHSS; hypertension; diabetes mellitus; smoking; drinking; hyperlipidaemia; renal markers (e.g. eGFR); HbA1c; hs‑CRP; homocysteine | Age; sex/gender; education; NIHSS; infarct volume; TOAST classification |
| Li et al. (2025) | Centrifuged at unknown × g at unknown temperature for unknown minutes. All samples were aliquoted and stored at a temperature of -80°C pending analysis. | NfL | (1) 22+ years old  (2) Ischaemic stroke (based on brain CT or MRI within 48 hours of symptom onset  (3) Systolic BP ranging from 140mmHg to 220 mmHg | (1) Systolic BP of ≥ 220 mmHg or diastolic BP of ≥ 120 mmHg  (2) Acute myocardial infarction (heart attack) or unstable angina or severe heart failure  (3) Aortic dissection, atrial fibrillation, cerebrovascular stenosis, resistant hypertension, deep coma | All patients suffered ischemic strokes  Thrombotic (CG1 103/157, CG2 104/154, CG3 101/149, CG4 102/162)  Embolic (CG1 2/157, CG2 8/154, CG3 44/149, CG4 58/162)  Lacunar (CG1 52/157, CG2 43/154, CG3 44/149, CG4 58/162)  CG1 = Low NfL (≤131.0 pg/mL) Anti-hypertensive treatment  CG2 = Low NfL (≤131.0 pg/mL) Control  CG3 = High NfL (>131.0 pg/mL) Anti-hypertensive treatment  CG4 = High NfL (>131.0 pg/mL) Control | Education; NIHSS; hypertension; diabetes mellitus; smoking; drinking; hyperlipidaemia; renal markers (e.g. eGFR); history of coronary heart disease; family history of stroke; BMI | No covariate adjustment reported |
| Mao et al. (2020) | 3ml of venous fasted blood was collected.   Centrifuged at 3000 × g at unknown temperature for 10 minutes.  Stored at -80°C. | Aβ42 | (1) Acute ischaemic stroke (within 7 days before admission)  (2) Right-handed  (3) New cases (no previous history of stroke) | (1) Aphasia, severe hearing or visual impairment  (2) Thyroid disease and other endocrine diseases  (3) Severe condition after cerebral infarction and unconsciousness unable to cooperate with examination and cognitive assessment  (4) Patients with consciousness obstacle prior to the illness onset [the National Institutes of Health Stroke Scale (NIHSS) > 10] or other diseases associated with cognitive impairment in patients  (5) Suffering from abnormal function of the heart, lungs, liver or systemic disease, estimated survival < 1 year  (6) Have a history of mental illness or behavioural disorders | Ischaemic stroke:  Large-artery atherosclerosis, CG1=39 CG2=58  Cardioembolism, CG1=6 CG2=8  Small-artery occlusion (lacunar), CG1=21 CG2=37  Other determined, CG1=5 CG2=9  Undetermined, CG1=1 CG2=4  CG1 = PSCI  CG2 = Non-PSCI | Education; NIHSS; hypertension; diabetes mellitus; smoking; drinking; hyperlipidaemia; renal markers (e.g. eGFR); BMI; systolic and diastolic blood pressure; atrial fibrillation | Age; sex/gender; education; BMI; smoking; drinking; history of diseases; NIHSS; LDL cholesterol; triglycerides |
| Peng et al. (2021) | Centrifuged at 10,000 × g at unknown temperature for 5 minutes. | NfL | (1) Age >18 years old  (2) First time clinically significant ischemic stroke (It is unclear from the study what is considered 'clinically significant')  (3) Brain MRI scan performed in Massachusetts General Hospital (MGH) or Brigham Women's Hospital (BWH) within 1 poststroke month  (4) No previous history of neuropsychiatric diseases, including epilepsy, cerebral vascular abnormalities (arteriovenous malformations or aneurysm), or brain trauma  (5) Serum sample collected and stored on admission  (6) Length of staying for inpatient rehabilitation >7 days  PLEASE NOTE: There is no mention of exclusion of revious history of cognitive impairment / dementia.  30 age-matched stroke-free patients admitted to SRH without neurologic diseases were included as controls | Not specifically stated | Ischaemic stroke  TOAST aetiology not stated | NIHSS; mRS (back‑calculated from FIM motor scores); hypertension; diabetes mellitus; smoking; time from stroke onset; infarct volume; WMH volume; length of hospital stay; coronary heart disease; thrombolysis (tPA); BMI | WMH volume; infarct volume; mRS |
| Sanchez et al. (2024) | Samples were collected and processed within 24 hours; they were shipped on ice pack (not frozen prior to sample processing) overnight, where they were immediately processed upon receipt for plasma isolation.  Centrifuged at 2000 × g at 4 °C for 15 minutes, and then stored at -80°C until processing. | Aβ (40 and 42), GFAP, NfL, p-tau181 | (1) The CVD group included participants who had experienced a mild to moderate ischaemic stroke event documented on MRI or computed tomography ≥ 3 months before enrolment and confirmed by radiologist, with or without cognitive impairment but with minimum Montreal Cognitive Assessment (MoCA) cut-off score of 18, and with any level of small vessel disease burden.  A healthy control (HC) group(n = 44), studied using the same ONDRI protocols in addition to brainAβ PET as part of the Brain-Eye Amyloid Memory (BEAM) study, was also included to be used as a reference for baseline plasma biomarker levels only and not for further analysis.  (FROM REF 16 within study)  GENERAL INCLUSION = English speaking, sufficient vision and hearing, minimum grade 8 education, Minimum MoCA 18, Reliable study partner (e.g., NOK) who is well informed about the patient, geographically accessible. | (1) Individuals with large vessel occlusion infarction causing severe neurological deficit  (2) Serious underlying disease other than the diseases in the ONDRI that could interfere with patient engagement for 3 years  (3) Poorly controlled diabetes or clinical diagnosis of serious eye disease  (4) History of cognitive impairment or dementia before the vascular event were excluded  (5) enrolled in a disease modifying therapeutic trial | Not reported | Education; mRS; renal markers (e.g. eGFR) | Age; sex; education; APOE4 carrier status |
| Shi et al. (2024) | 3500 × g at unknown temperature for 10 minutes. | Aβ42 | (1) Patients with acute cerebral infarction admitted to Bozhou People's Hospital  (2) Confirmed diagnosis through cranial CT or MRI and other imaging examinations  (3) Admitted to the hospital within 72 h of onset  (4) Right-handed  Control group: 34 patients with transient cerebral blood supply insufficiency or cerebral vascular stenosis admitted during the same period | (1) Cognitive impairment prior to onset  (2) Epilepsy, depression, Parkinson's disease, and other mental illness  (3) Severe organ diseases such as heart, lung, liver, and kidney  (4) History of alcohol or drug abuse | Not reported | Gender; Hypertension ; Hyperlipidemia | No covariate adjustment reported |
| Stokowska et al. (2021) | Unknown information on pre-processing of bloods. Stored in -70°C Aliquots. | NfL | (1) Aged 50 - 75 years  (2) Disability grade 2 or 3 on MRS  (3) Being in the late-phase of stroke (10 months- 5 years after an ischemic or haemorrhagic stroke with initial presence of hemispheric impact/symptoms)  (4) Subarachnoid haemorrhage with initial presence of hemispheric impact/symptoms  (5) Ability to understand written and oral information and instructions in Swedish  (6) Having housing  (7) Ability to travel to the place of intervention and evaluation  (8) No need for personal assistance in activities of daily living while participating in the treatment (going to the toilet, transport/transportation services for disabled, walking)  (9) Accepting allocation to either of the three groups which might mean accepting staying without any of the treatment procedures for one year | (1) Disability rated < 2 or > 3 on MRS  (2) An ischemic or haemorrhagic stroke or subarachnoid haemorrhage without hemispheric impact/symptoms  (3) Pronounced fear of horses or allergy constituting a risk for the patients to participate in the therapeutic riding  (4) Heart condition that constitutes a risk for the individual to participate in the interventions  (5) Non-controlled epileptic seizures constituting a risk for the patients to participate in the interventions  (6) Lack of cognitive and/or verbal ability or visual impairment that makes it difficult for the individual to understand instructions and/or evaluation  (7) Total paralysis of the affected arm  (8) Weight > 97 kg (to optimize safe horseback riding)  (9) Having more than a half-time employment  (10) Injury, disease or addiction that make the individual not suitable for the trial  (11) Participation in RGRM or therapeutic riding intervention < 10 months prior to inclusion  (12) Having an additional stroke within the past year (TIA is however accepted)  (13) Lack of willingness to participate in any of the treatment methods  (14) Living > 80 km from Gothenburg  (15) Dependent on transportation services for disabled across the community border which is not allowed according to the regulations | of haemorrhagic/ischaemic patients:  control = 12/24  R-MT = 11/28  H-RT = 14/26 | Education (years of schooling); NIHSS; mRS | Age; baseline Letter–Number Sequence score |
| Tang et al. (2018) | The samples were centrifuged at 2500×g at unknown temperature for 15 min and stored at -80 °C until needed for use. | Aβ (40 and 42), total tau | (1) Patients with a history of stroke who had been receiving regular follow-up evaluations at the Stroke Outpatient Clinic from November 2014 to November 2016 were recruited  (2) Clinical diagnoses of VD were made in accordance with the criteria for dementia. For the patients with AD, they must meet the diagnostic guidelines for dementia due to AD proposed by the NIA-AA workgroups in 2011 | (1) Pre-stroke significant functional disability (mRS > 1)  (2) Dementia (CDR > 1)  (3) Unwilling to receive blood drawing or signed informed consent were excluded  (4) Post-stroke moderate to severe aphasia, impaired consciousness, severe dysarthria  (5) History of depression  (6) GDS > 8  (7) Other neurodegenerative diseases | Not reported | mRS; hypertension; diabetes mellitus; smoking; hyperlipidaemia; coronary artery disease | No covariate adjustment reported |
| Wang et al. (2021a) | Centrifuged at unknown × g at unknown temperature for unknown minutes.  Stored at -80 degrees Celsius. | NfL | Patients were recruited at 1 month after the onset of AIS, when the patients crossed over the acute AIS stage and the symptoms were stable.  (1) Aged 60 yrs or older  (2) Self-reported cognitive impairment subsequent to an AIS event  (3) No cognitive impairment was observed from the Clinical Dementia Rating (CDR) scale  (4) Willing to participate in the study | (1) Cognitive impairment before AIS onset  (2) Family history of dementia  (3) Have psychiatric disorders (e.g., schizophrenia, bipolar disorders, depression) before AIS onset  (4) Cannot complete the cognitive test due to hearing, language, or communicating disabilities  (5) Have other severe neurological diseases that may affect circulating NfL levels (Parkinson's disease, Alzheimer's disease, traumatic brain injury, etc.)  (6) Refused to participate in the study | All patients had confirmed acute ischaemic stroke  Atherothrombotic - CG1: 85.49%, CG2: 79.59%  Cardioembolic - CG1: 5.88%, CG2: 8.16%  Lacunar - CG1: 4.31%, CG2: 12.24%  Unknown - CG1: 4.31%, CG2: 0.00%  CG1 = Stable group  CG2 = Progression group | Education; NIHSS; hypertension; diabetes mellitus; smoking; post‑stroke depression; post‑stroke anxiety; DWI hyperintensity volume; APOE4 carrier status; BMI; family history of stroke; antiplatelet and antithrombotic drug use; hypercholesterolaemia | Age; sex |
| Wang et al. (2021b) | Centrifugation for 20 min at 3,000g at room temperature, plasma (from the EDTA tube) was aliquoted.  The tubes were frozen locally at -80°C within 40 min after collection. | NfL | (1) ≥18 years with first ever acute ischaemic stroke of the anterior circulation within 24hr of symptom onset who were admitted to the General Hospital of Western Theatre Command between July 1, 2017 and December 31, 2019  (2) Patients were diagnosed according to the World Health Organisation criteria and confirmed using brain computed tomography (CT) or magnetic resonance imaging (MRI) | (1) Pre-existing cognitive impairment (clinical diagnosis or previous treatment or if the subject/caregiver reported progressive forgetfulness), mental illness or were unable to complete the cognitive assessments  (2) Other non-vascular causes of neural function defects (brain injury, Alzheimer's disease, Parkinson's disease, and other neurological diseases)  (3) Previous history of serious medical diseases, tumour, hepatitis or an autoimmune disease  (4) survived less than 3 months | Acute ischemic stroke (AIS) of the anterior circulation  TOAST:  Large-artery atherosclerosis (N= 928 54.78%)  Cardioembolism (N= 239 14.11%)  Small vessel occlusion (N= 145 9.09%)  OTHER (N= 68 4.01%)  Undetermined (305 18.01%) | Education; NIHSS; hypertension; diabetes mellitus; smoking; drinking; hyperlipidaemia; renal markers (e.g. eGFR); time to blood sampling; BMI; atrial fibrillation; hs‑CRP | Age; sex; education; NIHSS; infarct volume; TOAST classification |
| Zheng et al. (2023) | Centrifuged at 3000 × g at unknown temperature for 5 minutes. Plasma frozen at -80°C. | NfL | (1) 18+  (2) First episode of intracerebral haemorrhage within or at 24h were included (diagnosed by following the Guidelines for Diagnosis and Treatment of Cerebral Haemorrhage (2014)) | (1) Subarachnoid haemorrhage  (2) Haemorrhage transformation of cerebral infarction  (3) Subdural/extradural haematoma  (4) Neurodegenerative diseases, tumours  (5) No prior history of cognitive impairment, dementia or neuropsychiatric disease | All stroke types were intracerebral haemorrhage | NIHSS; mRS; hypertension; diabetes mellitus; smoking; Glasgow Coma Scale; cancer; ischaemic heart disease; cerebrovascular disease | No covariate adjustment reported |

# References

Chen HG, Wang M, Jiao AH, Tang GT, Zhu W, Zou P, et al. Research on changes in cognitive function, β-amyloid peptide and neurotrophic factor in stroke patients. *European Review for Medical and Pharmacological Sciences*. 2018;22(19): 6448–6455. <https://doi.org/10.26355/eurrev_201810_16057>.

Chi NF, Chao SP, Huang LK, Chan L, Chen YR, Chiou HY, et al. Plasma Amyloid Beta and Tau Levels Are Predictors of Post-stroke Cognitive Impairment: A Longitudinal Study. *Frontiers in Neurology*. 2019;10: 715. <https://doi.org/10.3389/fneur.2019.00715>.

Egle M, Loubiere L, Maceski A, Kuhle J, Peters N, Markus HS. Neurofilament light chain predicts future dementia risk in cerebral small vessel disease. *Journal of Neurology, Neurosurgery & Psychiatry*. 2021;92(6): 582–589. <https://doi.org/10.1136/jnnp-2020-325681>.

Ferrari F, Rossi D, Ricciardi A, Morasso C, Brambilla L, Albasini S, et al. Quantification and prospective evaluation of serum NfL and GFAP as blood-derived biomarkers of outcome in acute ischemic stroke patients. *Journal of Cerebral Blood Flow & Metabolism*. 2023;43(9): 1601–1611. <https://doi.org/10.1177/0271678X231172520>.

Gendron TF, Badi MK, Heckman MG, Jansen-West KR, Vilanilam GK, Johnson PW, et al. Plasma neurofilament light predicts mortality in patients with stroke. *Science translational medicine*. 2020;12(569): eaay1913. <https://doi.org/10.1126/scitranslmed.aay1913>.

Huang KL, Hsiao IT, Chang TY, Yang SY, Chang YJ, Wu HC, et al. Neurodegeneration and Vascular Burden on Cognition After Midlife: A Plasma and Neuroimaging Biomarker Study. *Frontiers in Human Neuroscience*. 2021;15. <https://doi.org/10.3389/fnhum.2021.735063>.

Huang LK, Chao SP, Hu CJ, Chien LN, Chiou HY, Lo YC, et al. Plasma Phosphorylated-tau181 Is a Predictor of Post-stroke Cognitive Impairment: A Longitudinal Study. *Frontiers in Aging Neuroscience*. 2022;14. <https://doi.org/10.3389/fnagi.2022.889101>.

Jiang L, Wang Z, Wang R, Li M, Zhang Y, Yang D. Plasma Neurofilament Light Chain Is Associated with Cognitive Impairment after Posterior Circulation Stroke. *Evidence-based Complementary and Alternative Medicine : eCAM*. 2022;2022: 2466982. <https://doi.org/10.1155/2022/2466982>.

Li H, Yang D, Liu S, Zhu Z, Shi M, Xu T, et al. Effects of early antihypertensive treatment on cognitive function in patients with acute ischemic stroke with different neurofilament light chain levels. *Journal of Stroke and Cerebrovascular Diseases*. 2025;34(2): 108206. <https://doi.org/10.1016/j.jstrokecerebrovasdis.2024.108206>.

Mao L, Chen XH, Zhuang JH, Li P, Xu YX, Zhao YC, et al. Relationship between β-amyloid protein 1-42, thyroid hormone levels and the risk of cognitive impairment after ischemic stroke. *World Journal of Clinical Cases*. 2020;8(1): 76–87. <https://doi.org/10.12998/wjcc.v8.i1.76>.

Peng Y, Li Q, Qin L, He Y, Luo X, Lan Y, et al. Combination of Serum Neurofilament Light Chain Levels and MRI Markers to Predict Cognitive Function in Ischemic Stroke. *Neurorehabilitation and Neural Repair*. 2021;35(3): 247–255. <https://doi.org/10.1177/1545968321989354>.

Sanchez E, Wilkinson T, Coughlan G, Mirza S, Baril AA, Ramirez J, et al. Association of plasma biomarkers with cognition, cognitive decline, and daily function across and within neurodegenerative diseases: Results from the Ontario Neurodegenerative Disease Research Initiative. *Alzheimer’s & Dementia*. 2024;20(3): 1753–1770. <https://doi.org/10.1002/alz.13560>.

Shi X, Zhang X, Ao J fu, Yang M. Correlation between Non-HDL-C/HDL-C and Aβ1-42 levels in cerebral infarction-related cognitive dysfunction. *Clinical Neurology and Neurosurgery*. 2024;245: 108503. <https://doi.org/10.1016/j.clineuro.2024.108503>.

Stokowska A, Bunketorp Käll L, Blomstrand C, Simrén J, Nilsson M, Zetterberg H, et al. Plasma neurofilament light chain levels predict improvement in late phase after stroke. *European Journal of Neurology*. 2021;28(7): 2218–2228. <https://doi.org/10.1111/ene.14854>.

Tang SC, Yang KC, Chen CH, Yang SY, Chiu MJ, Wu CC, et al. Plasma β-Amyloids and Tau Proteins in Patients with Vascular Cognitive Impairment. *NeuroMolecular Medicine*. 2018;20(4): 498–503. <https://doi.org/10.1007/s12017-018-8513-y>.

Wang JH, Huang J, Guo FQ, Wang F, Yang S, Yu NW, et al. Circulating Neurofilament Light Predicts Cognitive Decline in Patients With Post-stroke Subjective Cognitive Impairment. *Frontiers in Aging Neuroscience*. 2021a;13. <https://doi.org/10.3389/fnagi.2021.665981>.

Wang Z, Wang R, Li Y, Li M, Zhang Y, Jiang L, et al. Plasma Neurofilament Light Chain as a Predictive Biomarker for Post-stroke Cognitive Impairment: A Prospective Cohort Study. *Frontiers in Aging Neuroscience*. 2021b;13. <https://doi.org/10.3389/fnagi.2021.631738>.

Zheng P, Wang X, Chen J, Wang X, Shi SX, Shi K. Plasma Neurofilament Light Chain Predicts Mortality and Long-Term Neurological Outcomes in Patients with Intracerebral Hemorrhage. *Aging and disease*. 2023;14(2): 560–571. <https://doi.org/10.14336/AD.2022.21020>.
