# Supplementary material for: Dementia blood biomarkers in the context of post‐stroke cognitive outcomes: Systematic review and evidence synthesis
Source: Alzheimers Dement. 2026 Jul 6;22(7):e71653. doi: 10.1002/alz.71653 (PMC13337546; doi:10.1002/alz.71653)
Supplement: Supplementary file 5 — Supporting Information [file ALZ-22-e71653-s003.docx]

# Supplementary Material 4: Study and assay characteristics by biomarker group

| Biomarker (number of studies) | Age in years (Median [Range]) | Sample sizes (Median [Range]) | Stroke population no. of studies (Ischaemic / Mixed / Haemorrhagic) | Biomarker sampling methods (number of studies) |
| --- | --- | --- | --- | --- |
| NfL (11)^a^ | 65 (59 - 75) | 161 (26 – 1694) | 8 / 2 / 1 | SIMOA (10), electrochemiluminescence immunoassay (1) |
| Amyloid species (8)^b^ | 67 (59 – 75) | 72 (24 – 188) | 7 / 1/ 0 | IMR (4), ELISA (3), SIMOA (1) |
| Tau species (5)^c^ | 62 (59 – 75) | 61 (24 – 161) | 5 / 0 / 0 | Total tau: IMR (3) \| p-tau181: IMR (1), SIMOA (1) |
| GFAP (2)^d^ | 72 (69 – 75) | 99 (36 – 161) | 2 / 0 / 0 | SIMOA (2) |

ELISA = Enzyme-linked Immunosorbent Assay; GFAP = Glial Fibrillary Acidic Protein; IMR = Immunomagnetic Reduction; NfL = Neurofilament light; SIMOA = Single Molecular Array.

^a^ NfL study references: Egle et al. (2021); Ferrari et al. (2023); Gendron (2020); Jiang et al. (2022); Peng et al. (2021); Sanchez et al. (2024); Stokowska et al. (2021); Wang et al. (2021a); Wang et al. (2021b); Zheng et al. (2023).

^b^ Amyloid species references: Chen et al. (2018); Chi et al. (2019); Huang et al. (2021); Huang et al. (2022); Mao et al. (2020); Sanchez et al. (2024); Shi et al. (2024); Tang et al. (2018).

^c^ Tau species references: Chi et al. (2019); Huang et al. (2021); Huang et al. (2022); Sanchez et al. (2024); Tang et al. (2018).

^d^ GFAP species: Ferrari et al. (2023), Sanchez et al. (2024).

# References

Chen HG, Wang M, Jiao AH, Tang GT, Zhu W, Zou P, et al. Research on changes in cognitive function, β-amyloid peptide and neurotrophic factor in stroke patients. *European Review for Medical and Pharmacological Sciences*. 2018;22(19): 6448–6455. <https://doi.org/10.26355/eurrev_201810_16057>.

Chi NF, Chao SP, Huang LK, Chan L, Chen YR, Chiou HY, et al. Plasma Amyloid Beta and Tau Levels Are Predictors of Post-stroke Cognitive Impairment: A Longitudinal Study. *Frontiers in Neurology*. 2019;10: 715. <https://doi.org/10.3389/fneur.2019.00715>.

Egle M, Loubiere L, Maceski A, Kuhle J, Peters N, Markus HS. Neurofilament light chain predicts future dementia risk in cerebral small vessel disease. *Journal of Neurology, Neurosurgery & Psychiatry*. 2021;92(6): 582–589. <https://doi.org/10.1136/jnnp-2020-325681>.

Ferrari F, Rossi D, Ricciardi A, Morasso C, Brambilla L, Albasini S, et al. Quantification and prospective evaluation of serum NfL and GFAP as blood-derived biomarkers of outcome in acute ischemic stroke patients. *Journal of Cerebral Blood Flow & Metabolism*. 2023;43(9): 1601–1611. <https://doi.org/10.1177/0271678X231172520>.

Gendron TF, Badi MK, Heckman MG, Jansen-West KR, Vilanilam GK, Johnson PW, et al. Plasma neurofilament light predicts mortality in patients with stroke. *Science translational medicine*. 2020;12(569): eaay1913. <https://doi.org/10.1126/scitranslmed.aay1913>.

Huang KL, Hsiao IT, Chang TY, Yang SY, Chang YJ, Wu HC, et al. Neurodegeneration and Vascular Burden on Cognition After Midlife: A Plasma and Neuroimaging Biomarker Study. *Frontiers in Human Neuroscience*. 2021;15. <https://doi.org/10.3389/fnhum.2021.735063>.

Huang LK, Chao SP, Hu CJ, Chien LN, Chiou HY, Lo YC, et al. Plasma Phosphorylated-tau181 Is a Predictor of Post-stroke Cognitive Impairment: A Longitudinal Study. *Frontiers in Aging Neuroscience*. 2022;14. <https://doi.org/10.3389/fnagi.2022.889101>.

Jiang L, Wang Z, Wang R, Li M, Zhang Y, Yang D. Plasma Neurofilament Light Chain Is Associated with Cognitive Impairment after Posterior Circulation Stroke. *Evidence-based Complementary and Alternative Medicine : eCAM*. 2022;2022: 2466982. <https://doi.org/10.1155/2022/2466982>.

Li H, Yang D, Liu S, Zhu Z, Shi M, Xu T, et al. Effects of early antihypertensive treatment on cognitive function in patients with acute ischemic stroke with different neurofilament light chain levels. *Journal of Stroke and Cerebrovascular Diseases*. 2025;34(2): 108206. <https://doi.org/10.1016/j.jstrokecerebrovasdis.2024.108206>.

Mao L, Chen XH, Zhuang JH, Li P, Xu YX, Zhao YC, et al. Relationship between β-amyloid protein 1-42, thyroid hormone levels and the risk of cognitive impairment after ischemic stroke. *World Journal of Clinical Cases*. 2020;8(1): 76–87. <https://doi.org/10.12998/wjcc.v8.i1.76>.

Peng Y, Li Q, Qin L, He Y, Luo X, Lan Y, et al. Combination of Serum Neurofilament Light Chain Levels and MRI Markers to Predict Cognitive Function in Ischemic Stroke. *Neurorehabilitation and Neural Repair*. 2021;35(3): 247–255. <https://doi.org/10.1177/1545968321989354>.

Sanchez E, Wilkinson T, Coughlan G, Mirza S, Baril AA, Ramirez J, et al. Association of plasma biomarkers with cognition, cognitive decline, and daily function across and within neurodegenerative diseases: Results from the Ontario Neurodegenerative Disease Research Initiative. *Alzheimer’s & Dementia*. 2024;20(3): 1753–1770. <https://doi.org/10.1002/alz.13560>.

Shi X, Zhang X, Ao J fu, Yang M. Correlation between Non-HDL-C/HDL-C and Aβ1-42 levels in cerebral infarction-related cognitive dysfunction. *Clinical Neurology and Neurosurgery*. 2024;245: 108503. <https://doi.org/10.1016/j.clineuro.2024.108503>.

Stokowska A, Bunketorp Käll L, Blomstrand C, Simrén J, Nilsson M, Zetterberg H, et al. Plasma neurofilament light chain levels predict improvement in late phase after stroke. *European Journal of Neurology*. 2021;28(7): 2218–2228. <https://doi.org/10.1111/ene.14854>.

Tang SC, Yang KC, Chen CH, Yang SY, Chiu MJ, Wu CC, et al. Plasma β-Amyloids and Tau Proteins in Patients with Vascular Cognitive Impairment. *NeuroMolecular Medicine*. 2018;20(4): 498–503. <https://doi.org/10.1007/s12017-018-8513-y>.

Wang JH, Huang J, Guo FQ, Wang F, Yang S, Yu NW, et al. Circulating Neurofilament Light Predicts Cognitive Decline in Patients With Post-stroke Subjective Cognitive Impairment. *Frontiers in Aging Neuroscience*. 2021a;13. <https://doi.org/10.3389/fnagi.2021.665981>.

Wang Z, Wang R, Li Y, Li M, Zhang Y, Jiang L, et al. Plasma Neurofilament Light Chain as a Predictive Biomarker for Post-stroke Cognitive Impairment: A Prospective Cohort Study. *Frontiers in Aging Neuroscience*. 2021b;13. <https://doi.org/10.3389/fnagi.2021.631738>.

Zheng P, Wang X, Chen J, Wang X, Shi SX, Shi K. Plasma Neurofilament Light Chain Predicts Mortality and Long-Term Neurological Outcomes in Patients with Intracerebral Hemorrhage. *Aging and disease*. 2023;14(2): 560–571. <https://doi.org/10.14336/AD.2022.21020>.
