# Supplementary material for: Dementia blood biomarkers in the context of post‐stroke cognitive outcomes: Systematic review and evidence synthesis
Source: Alzheimers Dement. 2026 Jul 6;22(7):e71653. doi: 10.1002/alz.71653 (PMC13337546; doi:10.1002/alz.71653)
Supplement: Supplementary file 6 — Supporting Information [file ALZ-22-e71653-s001.docx]

# Supplementary Material 5: Risk of Bias consensus responses to each domain

## Bias due to confounding

| Study ID | Bias due to confounding | Bias due to confounding supporting text |
| --- | --- | --- |
| Chen et al. (2018) | Some concerns | Whilst the study authors did measure participant education level, the authors did not report adjusting MoCA or MMSE scores for this. The study did also not adjust for other covariates which have been suggested to impact blood biomarker levels (e.g., smoking, drinking, diabetes mellitus) however this is likely due to the studies small sample size (n=30) meaning adjustment was not feasible. |
| Chi et al, (2019) | Some concerns | Whilst the study authors did adjust MoCA scores based on participant education levels, they did not adjust for age or lesion volume despite obtaining this data. |
| Egle et al. (2021) | Low | Study authors adjusted for age, gender and premorbid IQ (NART) when investigating the predictive association between the change in markers and cognitive decline or dementia conversion. |
| Ferrari et al. (2022) | Some concerns | The authors did not collect information on participants education levels and therefore cognitive scores could not be adjusted to account for this. Due to the studies small sample size (n=36) it was not possible to account for confounding factors that could influence biomarker levels during most statistical analysis (e.g., smoking habit, renal function, BMI). Some confounding factors considered in multivariate analysis (“Patients’ age, pre-stroke mRS and chronic cerebrovascular burden (CCVB) identified by adapted Fazekas scale were selected a priori as clinical variables to be included as possible source of bias when evaluating these biomarkers”). |
| Gendron et al. (2020) | Some concerns | MMSE was adjusted for time from stroke to blood draw age at blood draw, sex, BMI, current smoking, hypertension, diabetes, and physical activity BUT education was NOT considered a covariate to be adjusted for in MMSE calculations - this is not standard. |
| Huang et al. (2021) | Low | The authors adjusted for age and education during partial correlation analysis. Partial correlation analysis was also adjusted for Geriatric Depression Scale (GDS) where possible. |
| Huang et al. (2022) | Low | ROC analysis was performed with all relevant covariates in mind when estimating performance of plasma biomarkers; with education, hypertension, and plasma p-tau181 as independent factors of PSCI. |
| Jiang et al. (2022) | Low | Study adjusted for main confounding factors (e.g., age, imaging outcomes, education levels, sex and baseline NIHSS) during regression analysis. |
| Li et al. (2025) | Low | Adjusted for most major confounding factors, however, did not adjust for stroke volume size (this may be because the data was not available). |
| Mao et al. (2020) | Low | Main confounding factors were considered for the regression model used and MoCA scores were adjusted for participant education levels. |
| Peng et al. (2021) | Some concerns | Despite education being a known confounding factor for cognitive scoring (e.g., MoCA), the authors did not collect any data on participant education levels. There was also no evidence of controlling for other co-variates in regression models. Additionally, it is important to note that it is very unclear how many days post-stroke blood samples were taken from patients (blood samples taken "on admission" and "on discharge" to/from hospital however this could range from a couple days to a year). |
| Sanchez et al. (2024) | Low | Study participation criteria meant that confounding factors did not influence results (English comprehension, visual acuity, and auditory acuity). The authors also adjusted cognitive outcomes for age, sex, and education. |
| Shi et al. (2024) | High | Authors did not make any adjustments for any covariates during regression modelling. |
| Stokowska et al. (2021) | Some concerns | Letter Number Sequence cognitive score was adjusted for age and baseline NIHSS. However, education has been considered a covariate in the odds ratio analysis but not in other analysis. It is also important to note that key patient demographics have not been recorded (e.g., CKD, BMI, hypertension, hyperlipidaemia, ethnicity) and thus could not be considered as confounding factors during analysis. |
| Tang et al. (2018) | Some concerns | Whilst the study did report adjusting for some confounding factors during the odds ratio analysis, education was not considered as a covariate potentially leading to some bias. |
| Wang et al. (2021a) | Low | Various covariates (e.g., demographic and imaging factors) were accounted for in regression models. |
| Wang et al. (2021b) | Low | Study accounted for all major known confounding factors in their regression analysis. |
| Zheng et al. (2023) | High | It is important to note that the Control group appear much younger than the stroke group with a difference between of 13 years (Age, years (minimum, maximum): Control group 46 (25, 79), ICH patients 59 (27, 84)). The authors have not mentioned whether statistical tests were done to test whether the age between the two groups were statistically different. Also, the authors did not measure the education levels/years of participants and therefore could not make the appropriate adjustments to cognitive data. No mention of MoCA scores being adjusted for age or sex (demographics which were collected). Ethnicity ratios of patient population not mentioned. |

## Bias in selection of participants into the study

| Study ID | Bias in selection of participants into the study | Bias in selection of participants into the study supporting text |
| --- | --- | --- |
| Chen et al. (2018) | Some concerns | The study did not excluded patients with cognitive impairment prior to stroke. It is also vague as to where patients were recruited from: "A total of 30 patients with acute stroke treated in our hospital from June 2015 to September 2016...". |
| Chi et al, (2019) | Some concerns | Due to harsh exclusion criteria, most participants had small lesions and small vessel disease; therefore, results may not represent patient population. Also, 80% of the studies participants are male, again reducing the generalisability of the results. |
| Egle et al. (2021) | Low | Clear eligibility criteria and recruitment from a well-defined population. This is also a multisite study, with patients recruited from 3 different hospitals within South London. |
| Ferrari et al. (2022) | Low | Small sample size (n=36) of mainly Caucasian heritage and low mRS. Taken together, study sample likely will not represent patient population. However, it was concluded that it is unlikely to be done purposefully by the authors. |
| Gendron et al. (2020) | Some concerns | It is unclear whether participants with a history of cognitive impairment pre-stroke were included in this study. The participants also had a low ethnic diversity and were mainly of Caucasian descent. |
| Huang et al. (2021) | Some concerns | Participants were mainly males with minor strokes (patient NIHSS scores were 2.6 ± 1.4 points at stroke onset and 1.7 ± 1.1 points 3 months post-stroke), this may affect the generalisability of the results. Stroke patients were also recruited around 3 months after stroke incidence however the timeline could vary between participants and average time post stroke is not disclosed. |
| Huang et al. (2022) | Low | Patient population is mild stroke patients with small vessel disease, limiting the generalisability of the results. However, it was concluded that it is unlikely to be done purposefully by the authors. Ethnicity of patients not recorded. |
| Jiang et al. (2022) | Low | Clear inclusion and exclusion criteria. |
| Li et al. (2025) | Some concerns | The authors did not exclude participants with pre-existing cognitive impairment or dementia. |
| Mao et al. (2020) | High | The authors did not exclude participants with pre-existing cognitive impairment or dementia. The authors only included right-handed patients in the study however did not provide an explanation for this. Many of the studies participants are 'illiterate' however it is not disclosed how participant education levels were measured and it is also unknown how written informed consent was gained from these patients. |
| Peng et al. (2021) | High | Authors have recruited patients with "clinically significant ischaemic stroke" however it is unclear what criteria makes up "clinically significant" and therefore could introduce bias in the selection of participants. There is also no exclusion criteria for patient with a previous history of cognitive impairment / dementia. |
| Sanchez et al. (2024) | Low | Clear inclusion and exclusion criteria. |
| Shi et al. (2024) | Some concerns | No mention of exclusion for dementia diagnosis however the authors have excluded for previous history of cognitive impairment. Control group consisted of inpatients suffering from cerebral vascular stenosis and transient cerebral blood supply insufficiency, but it is not explained as to why this was an appropriate control group. |
| Stokowska et al. (2021) | High | Strict eligibility criteria of modified Rankin Scale score 2-3 could arguably limit the generalisability of findings. There is also large variability in the days post stroke of participants (ranging from 10 months to 5 years). No mention of patient ethnicity. No exclusion of individuals who have had previous cognitive impairment or dementia diagnosis. |
| Tang et al. (2018) | Some concerns | Single site study and thus the possibility of case selection bias cannot be excluded. The study has also not stated/recorded important patient demographics such as volume of stroke lesion, stroke type, and stroke aetiology. There is no explicit exclusion criteria for study participants with a previous history of cognitive impairment. There was large variability in the timeline between patients with some patients recruited up to 6 years post stroke - this could affect the assessment of stroke biomarker levels. |
| Wang et al. (2021a) | Low | Clear inclusion and exclusion criteria. |
| Wang et al. (2021b) | Some concerns | Single site study and thus the possibility of case selection bias cannot be excluded. No reports of ethnicity of patient population. Exclusion of patients with aphasia may affect the generalisability of results. |
| Zheng et al. (2023) | Low | Clear inclusion and exclusion criteria. |

## Bias in classification of interventions / exposures

| Study ID | Bias in classification of interventions / exposures | Bias in classification of interventions / exposures supporting text |
| --- | --- | --- |
| Chen et al. (2018) | Some concerns | Measurement of biomarker levels were well described, however, patient blood biomarkers were measured before and after "treatment" but it is never explicitly defined how "treatment" is classified. |
| Chi et al, (2019) | Some concerns | Standardised assay described however undefined temperature used for processing of bloods. |
| Egle et al. (2021) | Some concerns | There is no description of how the blood samples were stored, nor pre-processed before the analysis. The method of analysis, however, was clearly defined. |
| Ferrari et al. (2022) | Low | Standardised assay or biomarker measurement clearly described and validated. |
| Gendron et al. (2020) | Low | Standardised assay or biomarker measurement clearly described and validated. |
| Huang et al. (2021) | Some concerns | Undefined temperature used for processing of bloods. However, other measurements, such as neuroimaging, were clearly defined. |
| Huang et al. (2022) | Low | Standardised assay or biomarker measurement clearly described and validated. |
| Jiang et al. (2022) | Low | Standardised assay or biomarker measurement clearly described and validated. |
| Li et al. (2025) | Some concerns | Blood sample processing was not clearly outlined and missing details such as centrifugation temperature, time, and speed. |
| Mao et al. (2020) | Some concerns | Whilst ELISA was stated, more information on the technique could have been provided (lower limit of detection for example). Blood samples were also centrifuged at an unknown temperature. |
| Peng et al. (2021) | Some concerns | Undefined temperature used for processing of bloods. Also, the paper does not report the timeline of when blood samples were taken after the stroke event (bloods taken from admission instead) and the timeline could vary between participants (e.g., what if a patient has had a long lie before hospital admission?). |
| Sanchez et al. (2024) | Low | Standardised assay or biomarker measurement clearly described and validated. |
| Shi et al. (2024) | Some concerns | Undefined temperature used for processing of bloods. Samples were also stored in unknown conditions until analysis. The study did not detail what ELISA technique was used, nor the units for any biomarker levels (please see Table 2). |
| Stokowska et al. (2021) | Some concerns | Bloods processing method not fully described and excludes important information regarding centrifugation settings. |
| Tang et al. (2018) | Some concerns | Undefined temperature used for processing of bloods. |
| Wang et al. (2021a) | High | Blood sample processing method / centrifugation settings not fully described: “Blood was sampled at the first face-to-face interview, and serum was separated within 30 min after sampling and stored at −80◦C until further analysis”. Also, the studies patients were recruited at least 1 month post stroke however the authors have not stated the variability of when the blood samples were taken post stroke between participants. |
| Wang et al. (2021b) | Some concerns | The authors state in their discussion that "the centrifugal operation process was slightly different from the current standard guidelines, and the results should be further validated in future studies." (centrifugal operation process: "centrifugation for 20 min at 3,000 g at room temperature, plasma (from the EDTA tube) was aliquoted"). It is unclear why the research team decided on those particular centrifuge settings. |
| Zheng et al. (2023) | Some concerns | Measurements were defined well but there was a small omission of what temperature the blood samples were centrifuged at. All stratification criteria were explained, and biomarker measurement was well-defined. |

**Bias due to missing data (unreported)**

| Study ID | Bias due to missing data (unreported) | Bias due to missing data (unreported) supporting text |
| --- | --- | --- |
| Chen et al. (2018) | Some concerns | There were 30 participants stated to be part of the analysis, but from Figure 3A, the data of 28 participants data were used. There is mention as to why this data was missing / the potential exclusion or attrition of 2 participants. |
| Chi et al, (2019) | Some concerns | In the methods, it is stated that blood samples were collected from patients at baseline (up to 7 days post stroke) however the study has presented little data from this sample. The authors also did not explain why this set of data was not analysed. |
| Egle et al. (2021) | Low | Low attrition, with transparent reporting and appropriate handling e.g., “Due to a small sample size, observations coming from follow-up time point 4 and 5 were removed”, "One outlier observation at baseline was excluded as being nearly 30 times higher than any of the patient's follow-up NfL values. This patient did not show any clinical aspects that could explain such a high value as being 63 years old, having been diagnosed with diabetes and having suffered a clinical stroke 7 years ago.”, and participants with missing data had their data estimated using maximum likelihood estimation i.e. the study accounted for missing patient data. |
| Ferrari et al. (2022) | Low | Some missing data (dropouts and missing longitudinal blood data) however the reason for this has been explained (COVID, patient deaths etc) and is unlikely due to bias. |
| Gendron et al. (2020) | Low | Low attrition, with transparent reporting and appropriate handling - e.g., "We performed additional examination of the ability of NFL to independently predict 3- and 6-month mRS in two ways, similar to the analysis of the Mayo Clinic discovery series. First, the multivariable binary logistic regression models were additionally adjusted for NIHSS at initial evaluation (NIHSS at blood draw was not examined here due to missing data in the Yale ICH study series and lack of any data in the longitudinal ICH series). " |
| Huang et al. (2021) | Low | No attrition (cross-sectional study). Additionally, there is no apparent missing data. |
| Huang et al. (2022) | Low | Low attrition, with transparent reporting and appropriate handling. |
| Jiang et al. (2022) | Low | Low attrition, with transparent reporting and appropriate handling - "Twelve patients died within three months, eight patients had other central nervous system diseases, nine patients were unable to complete the cognitive assessments, seven patients withdrew consent or were lost to follow-up, and ten patients missed blood samples." |
| Li et al. (2025) | No information | No missing data reported. |
| Mao et al. (2020) | Low | Low attrition, with transparent reporting and appropriate handling. |
| Peng et al. (2021) | No information | No attrition apparent / reported. |
| Sanchez et al. (2024) | Low | Any missing data has transparent reporting and appropriate handling. It is key to note that the authors have reported "While age, sex, years of education, and plasma biomarker levels were not significantly different in participants with and without follow-ups, the latter group showed greater impairments in cognitive function: participants without any follow-ups had significantly worse attention and working memory (t = -2.07, P = 0.044), executive function (t = -2.32,P = 0.025), and visuospatial function (t = -2.10, P = 0.036) at baseline" and whilst the authors have addressed the impact, this is still likely to affect the bias of the results. |
| Shi et al. (2024) | No information | No attrition with no missing data reported (this is a cross-sectional study). |
| Stokowska et al. (2021) | Some concerns | Whilst some missing data was reported in the methodology and handled appropriately, Table 1 baseline characteristics do not add up and may suggest that the authors have excluded 2 patients - this is not reported within the text. |
| Tang et al. (2018) | Low | Transparent reporting and appropriate handling, e.g., prior to inclusion in the study, any individual that had a missing value for a primary clinical variable was excluded, so all participants should have all data present. |
| Wang et al. (2021a) | Low | Patient attrition was documented on Figure 1 and no other missing data was reported. |
| Wang et al. (2021b) | Low | Transparent reporting and appropriate handling of attrition. No other missing data was observed and hence reported. |
| Zheng et al. (2023) | Low | Transparent reporting and appropriate handling of missing data (e.g., medical data was unavailable for three patients, and blood samples were unavailable for eight others and therefore these patients were excluded). |

**Bias in measurement of outcomes**

| Study ID | Bias in measurement of outcomes | Bias in measurement of outcomes supporting text |
| --- | --- | --- |
| Chen et al. (2018) | Some concerns | Validated cognitive tests (MoCA and MMSE) were used, however, blinding of assessors has not been mentioned. Patients had data collected before and after "treatment" but it is It never defined what "treatment" is / describes. |
| Chi et al, (2019) | Low | Blinding mentioned in methods for the processing bloods, assessment of cognition, and the gathering of neuroimaging data metrics. |
| Egle et al. (2021) | Low | Authors stated that the individual performing NfL was blinded to dementia outcome measures. Neurologist and clinical neuropsychologists performing cognitive assessments were also blinded to the imaging and risk factor information. |
| Ferrari et al. (2022) | Some concerns | Blinded investigators were used for the assessment of neurological deficits and imaging outcomes, however, was NOT present for blood biomarker analysis. It is also important to note that the cognitive FIM is usually used to measure cognitive independence (related to activities of daily living) and may not capture the same cognitive domains as the MoCA or MMSE, which are more targeted towards MCI patient populations. |
| Gendron et al. (2020) | Low | NfL measurement was done in a blinded manner; imaging outcomes were also blinded to NfL concentrations. No blinding has been mentioned for the performance of cognitive assessments; however, it is important to note that cognitive assessments were performed at the time of consent and thus it is unlikely that NfL data was known / collected yet. |
| Huang et al. (2021) | Some concerns | The authors have not mentioned whether assessors were blinded to blood biomarker results during cognitive assessments (MMSE and CDR). There is also no mention of blinding for MRI data metrics either. |
| Huang et al. (2022) | Low | Imaging ratings were performed by an investigator blinded to the clinical details. Both neuropsychologists performing cognitive tests were blinded to patient's plasma biomarker data. Validated outcome measures were used for cognition, imaging and bloods. Whilst not a major cause for concern, it could be argued that a CDR-SB of 0.5 may be a harsh cut-off score for PSCI. |
| Jiang et al. (2022) | Low | Raters of imaging outcomes were blinded to clinical and laboratory outcomes. Psychiatrists were blind to the clinical data when performing the MoCA. |
| Li et al. (2025) | Low | Trained neurologists were blinded to the treatment assigned when conducting the MMSE. They also conducted a sensitivity analysis to account for covariates and the initial harsh MMSE criteria for PSCI. |
| Mao et al. (2020) | Some concerns | No mention of blinding with assessors performing the MoCA test. Also, for the 'illiterate' population (n=50), if they are in-fact 'illiterate', the MoCA Basic should have been used instead of the regular MoCA. |
| Peng et al. (2021) | High | There was no mention of blinding of assessors for the collection of cognitive and biomarker data. The authors collected cognitive date (FIM cognitive sub-score) on discharge; discharge days post stroke will be highly variable between participants, and it is therefore important to record this variability. Additionally, the paper never disclosed what the median FIM cognitive score was. Whilst the FIM is a good tool for measuring functional independence, the FIM cognitive subdomain is primarily used as a tool for "how cognition affects communication, safety, and independence", rather than assessing cognition / screening for mild cognitive impairment (e.g., a patient may be cognitive well whilst needing some assistance with activities of daily living). The appropriateness of using the FIM as an assessment for PSCI could therefore be questioned. |
| Sanchez et al. (2024) | Low | Cognitive outcome was measured well and explained in depth through referencing the appropriate paper for further details. Blinding of bloods was also described. |
| Shi et al. (2024) | Some concerns | No blinding mentioned by authors for neither the measurement or MMSE or the biomarker analysis. |
| Stokowska et al. (2021) | Some concerns | This study was a single-blinded, three-armed, randomised trial (patients were blinded however the researchers are not). This could introduce bias to the results of the cognitive assessments if the researchers conducting these assessments are not blinded. |
| Tang et al. (2018) | Low | The authors have stated that the CDR, MoCA, and MMSE scores were evaluated by the study nurses who were blinded to the blood testing results. |
| Wang et al. (2021a) | Some concerns | Cognitive test used was appropriately referenced and validated, but this test does lack face-face elements. No mentioning of blinding either when collecting any of the other data metrics. |
| Wang et al. (2021b) | Low | Validated cognitive tests (e.g., MoCA, MMSE) used with blinding of assessors. |
| Zheng et al. (2023) | Some concerns | No mention of blinding during the measurement of any outcomes (imaging/bloods/clinical status). Blood samples at days 7 and 14 post-ICH were only available for 41 patients which is a relatively small sample size. Validated cognitive test used for data collection (MoCA). |

**Bias in selection of the reported results**

| Study ID | Bias in selection of the reported results | Bias in selection of the reported results supporting text |
| --- | --- | --- |
| Chen et al. (2018) | High | No numerical values for average level of each biomarker in patient blood samples were reported, only statistical significance. In addition, ONLY statistically significant values were presented. This could suggest selective outcome reporting or suppression of possible negative findings. It is also important to note that many key patient demographics have not been measured/reported (e.g., mRS, NIHSS, location of stroke, stroke volume, aetiology, ethnicity….). Thus, making it extremely unclear as to what patient group these results are associated with. Additionally; despite collecting the MoCA scores of their patients (see methods), no analysis was done to investigate correlations with MoCA scores and blood biomarkers; the authors have not reported the results of whether there were correlations between plasma tau and cognition; and the reported correlation values are present in R^2 (this is not standard). |
| Chi et al, (2019) | Some concerns | Despite collecting baseline (up to 7 days post stroke) bloods (please see methods), it does not appear that the authors have not analysed these results. The authors also did not explain why this set of data was not analysed. It seems very strange that whilst bloods have been gathered at this time point, no data has been presented in the results section. |
| Egle et al. (2021) | Low | All outcomes reported as per protocol or methods section. |
| Ferrari et al. (2022) | Low | All outcomes reported as per protocol or methods section. |
| Gendron et al. (2020) | Low | All outcomes reported as per protocol or methods section. |
| Huang et al. (2021) | Low | All outcomes reported as per protocol or methods section. |
| Huang et al. (2022) | Low | All outcomes reported as per protocol or methods section. |
| Jiang et al. (2022) | Low | All outcomes reported as per protocol or methods section. |
| Li et al. (2025) | Low | All outcomes reported as per protocol or methods section. |
| Mao et al. (2020) | Some concerns | The progression of PSCI was compared with baseline and sorted into 3 groups based on changes in MoCA scores: aggravation, stable, and improvement. However, it is not known / it is very unclear at what time point progression was categorised (i.e., did they compare the baseline MoCA to the 12 month or 6-month MoCA score to determine whether someone was stable, aggravated, or improved). Could assume 12 months, however, what about dropouts? OR what if a patient showed aggravation at 3 months but cognitively improved at 1 year post stroke? |
| Peng et al. (2021) | Some concerns | Reporting of some important data values were not present. This included: (1) Patient were categorised into 2 groups: 'high' or 'low' median cognitive FIM subscore. Cut off scores for this categorisation have not been stated. (2) How long after a stroke was someone admitted into the hospital. (3) The variability in time from admission to discharge between patients. |
| Sanchez et al. (2024) | Low | Whilst all statistical outcomes (including non-significant results) were reported, the numerical values were not reported in the primary paper nor where they in the supplementary material. |
| Shi et al. (2024) | Low | All outcomes reported as per protocol or methods section. |
| Stokowska et al. (2021) | Some concerns | Education only considered as a covariate for SOME cognitive data analysis (odds ratio analysis). This could suggest the selective inclusion of a covariate. |
| Tang et al. (2018) | High | Whilst present in the scatter plot, the authors have not presented a quantitative value for the significant differences between Aβ-42, tau, and Aβ-40 levels between AD among the four groups (i.e. they did not provide the mean/median values for this comparison). Also, the number of participants included in the multivariate regression analysis has not been stated; given that the authors removed participants from analysis if that had missing data, this information is important to disclose. |
| Wang et al. (2021a) | Low | All outcomes reported as per protocol or methods section. |
| Wang et al. (2021b) | Low | All outcomes reported as per protocol or methods section. |
| Zheng et al. (2023) | Some concerns | Demographic numbers for certain variables (e.g., haemorrhagic features and NIHSS) are not accurate as they do not match sample size, please see Table 1. It may be that these patients did not have this data available however it is important to note that the omission of several patients could affect the results. |
